# A New Troodontid Theropod Dinosaur from the Lower Cretaceous of Utah

**DOI:** 10.1371/journal.pone.0014329

**Published:** 2010-12-15

**Authors:** Phil Senter, James I. Kirkland, John Bird, Jeff A. Bartlett

**Affiliations:** 1 Department of Biological Sciences, Fayetteville State University, Fayetteville, North Carolina, United States of America; 2 Utah Geological Survey, Salt Lake City, Utah, United States of America; 3 College of Eastern Utah Prehistoric Museum, Price, Utah, United States of America; 4 College of Eastern Utah, Price, Utah, United States of America; Raymond M. Alf Museum of Paleontology, United States of America

## Abstract

**Background:**

The theropod dinosaur family Troodontidae is known from the Upper Jurassic, Lower Cretaceous, and Upper Cretaceous of Asia and from the Upper Jurassic and Upper Cretaceous of North America. Before now no undisputed troodontids from North America have been reported from the Early Cretaceous.

**Methodology/Principal Findings:**

Herein we describe a theropod maxilla from the Lower Cretaceous Cedar Mountain Formation of Utah and perform a phylogenetic analysis to determine its phylogenetic position. The specimen is distinctive enough to assign to a new genus and species, *Geminiraptor suarezarum*. Phylogenetic analysis places *G. suarezarum* within Troodontidae in an unresolved polytomy with *Mei*, *Byronosaurus*, *Sinornithoides*, *Sinusonasus*, and *Troodon* + (*Saurornithoides* + *Zanabazar*). *Geminiraptor suarezarum* uniquely exhibits extreme pneumatic inflation of the maxilla internal to the antorbital fossa such that the anterior maxilla has a triangular cross-section. Unlike troodontids more closely related to *Troodon*, *G. suarezarum* exhibits bony septa between the dental alveoli and a promaxillary foramen that is visible in lateral view.

**Conclusions/Significance:**

This is the first report of a North American troodontid from the Lower Cretaceous. It therefore contributes to a fuller understanding of troodontid biogeography through time. It also adds to the known dinosaurian fauna of the Cedar Mountain Formation.

## Introduction

The family Troodontidae and its sister family Dromaeosauridae make up the clade Deinonychosauria [1]. Deinonychosauria, the sister taxon to Avialae, is a member of the theropod clade Coelurosauria [1]. Members of Troodontidae are gracile bipeds with an enlarged ungual on the second toe of each foot. They have been reported from the Upper Jurassic and Lower Cretaceous of China [2]–[5], the Lower and Upper Cretaceous of Mongolia [6]–[9], and the Upper Cretaceous of North America [10]. A tooth from the Upper Jurassic Morrison Formation of Utah has been referred to Troodontidae [11], but this assignment has been questioned [1]. A coelurosaurian skeleton from the Morrison Formation of Wyoming is possibly troodontid [12], but to date a description has not been published. Before now, no description has been published of an undisputed troodontid from North American sediments that predate the Late Cretaceous. Here we report the discovery of a troodontid maxilla from the basal Cedar Mountain Formation (Lower Cretaceous, Barremian?) of Utah.

## Methods

### Phylogenetic Analysis

We entered data from the new specimen into a phylogenetic data matrix of Coelurosauria from a recent study [13] (Appendix S1). All characters were left unordered. The non-coelurosaurian theropods *Dilophosaurus*, *Allosaurus*, and *Sinovenator* were used as outgroups. The matrix from that study [13] was based on one published previously [14] but included updates and corrections that are delineated and justified elsewhere [13]. Phylogenetic analysis was performed with PAUP 4.0 for Windows [15]. A heuristic search with 1000 random addition-sequence replicates was performed, with no limit to “maxtrees.”

## Results

### Systematic Paleontology

**Systematic hierarchy:**

Dinosauria Owen, 1841 [16]

Saurischia Seeley, 1887 [17]

Theropoda Marsh, 1881 [18]

Coelurosauria von Huene, 1914 [19]

Troodontidae Gilmore, 1924 [20]

*Geminiraptor* gen. nov.

urn: lsid:zoobank.org:act:57057FD5-AB25-43F1-A9AA-C3C2E51B0C9F

*Geminiraptor suarezarum* sp. nov.

urn:lsid:zoobank.org:act:4B950765-6016-4FC8-96ED-2219112C6E48

#### Holotype

**Figure 1:**
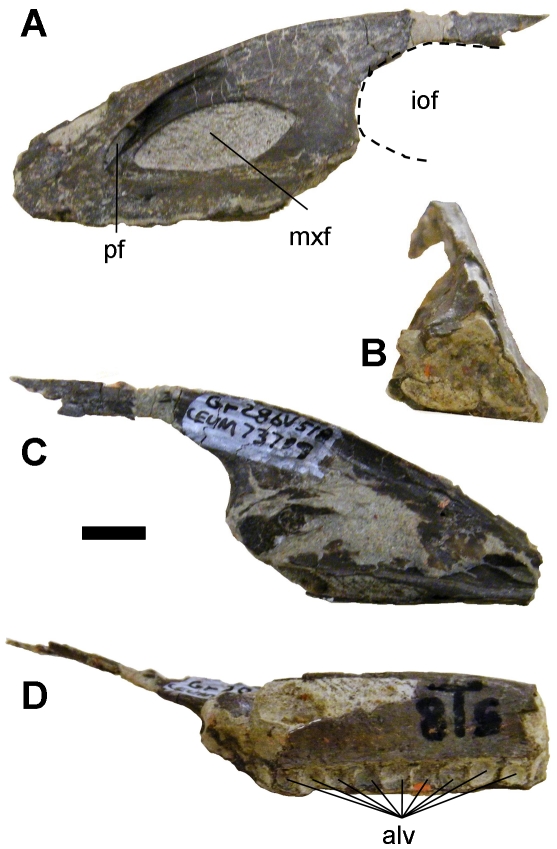
Maxilla of *Geminiraptor suarezarum.* (A)—Lateral view. (B)—Cranial view. (C)—Medial view. (D)—Ventral view. Scale bar = 10 mm. alv  =  dental alveoli, iof  =  internal antorbital fenestra, mxf  =  maxillary fenestra, pf  =  promaxillary fenestra.

The holotype specimen is CEUM (College of Eastern Utah Prehistoric Museum, Price Utah) 7319, a maxilla (Fig. 1).

#### Etymology

The species name refers to Drs. Celina and Marina Suarez, the twin geologists who discovered the Suarez site. The genus name is from the Latin *geminae* (“twins,” in honor of the Suarez sisters) and *raptor* (“one who seizes or takes by force,” a common part of deinonychosaurian genus names).

#### Locality and horizon

**Figure 2:**
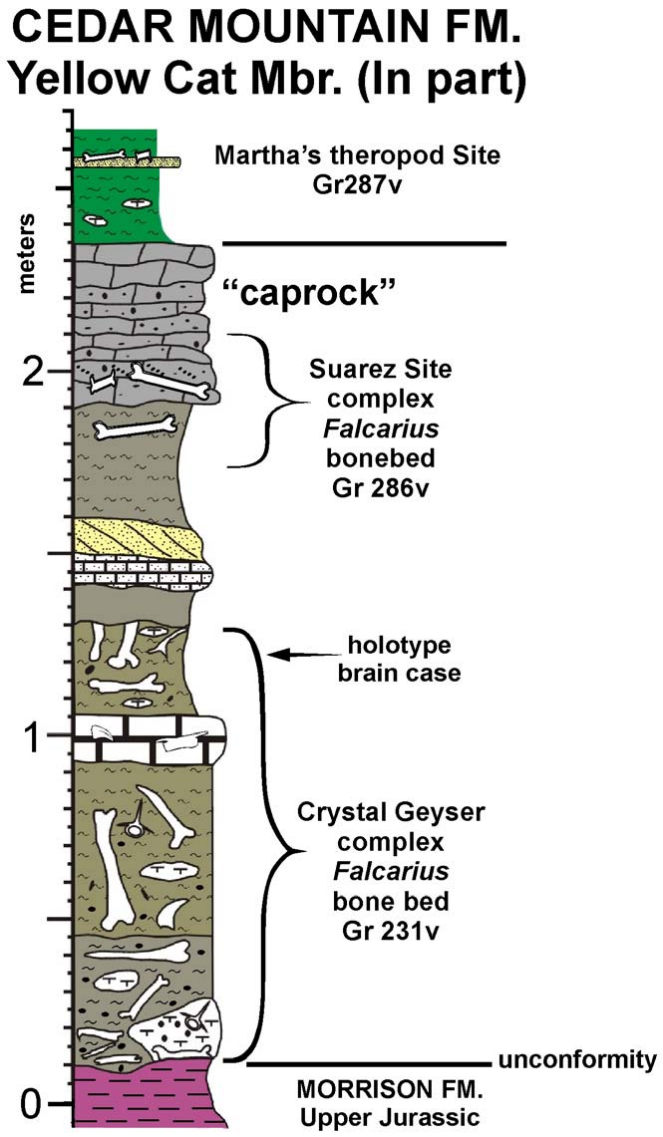
Stratigraphic setting of *Geminiraptor suarezarum*.

The specimen is from the Suarez site [21], a dinosaur bonebed (dominated by the therizinosauroid *Falcarius*) in the lower Yellow Cat Member (Lower Cretaceous: Barremian?) [22] of the Cedar Mountain Formation (Fig. 2). The site is in western Grand County, Utah.

#### Diagnosis

Troodontid possessing a maxilla with extensive pneumatization internal to the antorbital fossa, inflating the bone so that it has a triangular cross-section; a large, craniocaudally elongate maxillary fenestra; a promaxillary fenestra that is visible in lateral view; craniocaudally narrow promaxillary strut and interfenestral strut; small, square dental alveoli with bony septa between them.

### Specimen Description

**Figure 3:**
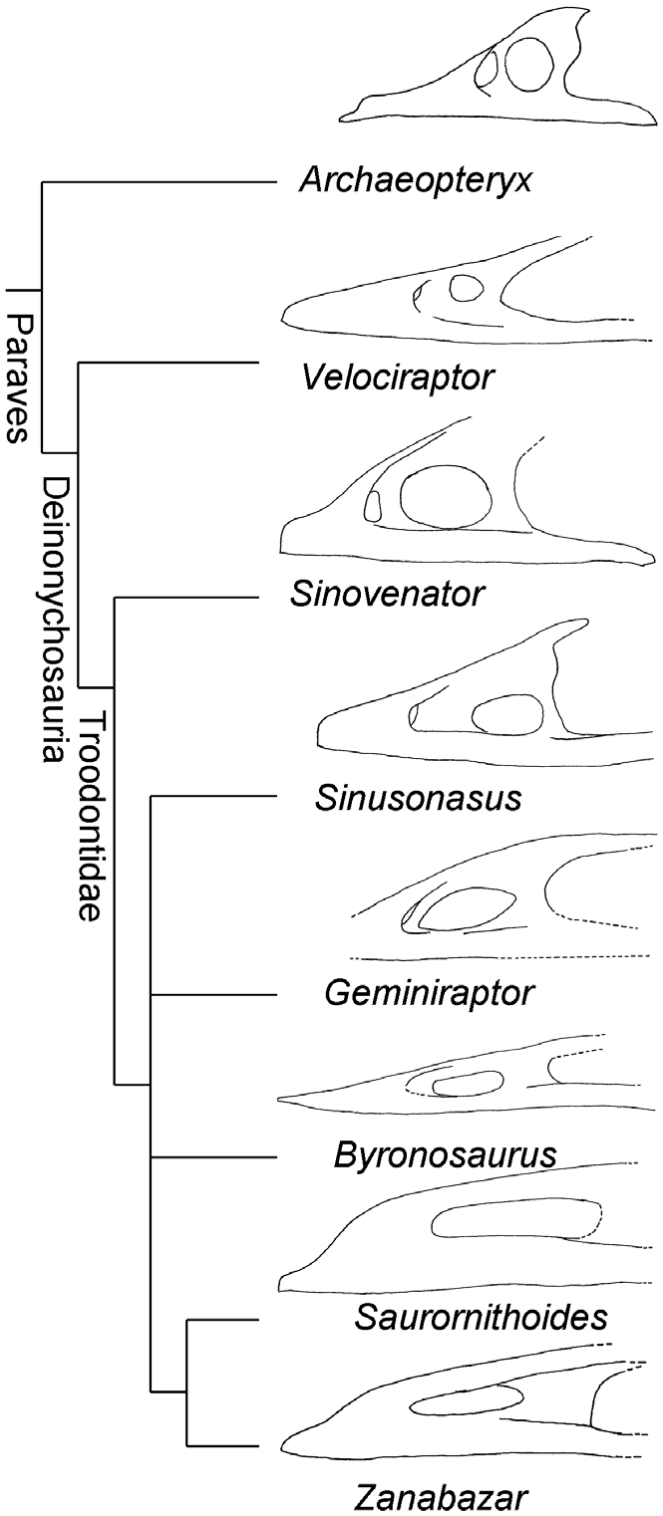
Comparison of the maxilla of *Geminiraptor suarezarum* to those of other paravians. Maxillae are drawn from photos by P.S. of AMNH (American Museum of Natural History, New York City, New York) FR 6515 (*Velociraptor*), IVPP V 12615 (*Sinovenator*), IVPP V 11527 (*Sinusonasus*), CEUM 7319 (*Geminiraptor*), IGM (Mongolian Institute of Geology, Ulaan Baatar, Mongolia) 100/983 (*Byronosaurus*), AMNH FR 6516 (*Saurornithoides*), and IGM 100/1 (*Zanabazar*); and reference 18 (*Archaeopteryx*).

The maxilla is craniocaudally long and dorsoventrally low. The process dorsal to the external antorbital fenestra is horizontal, as in the troodontids *Byronosaurus* [8], *Saurornithoides* [9], and *Zanabazar* [9] (Fig. 3). The cranial tip of the maxilla is missing, as is the portion of the bone ventral to the internal antorbital fenestra. The preserved portion of the maxilla is 95 mm long, 30 mm tall, and 20 mm wide at the sixth preserved alveolus. Due to preservation, it is not possible to determine whether the maxilla participated in the border of the external naris.

The antorbital fossa occupies a large portion of the lateral surface of the maxilla cranial to the internal antorbital fenestra, as in other troodontids [2], [4], [8], [9], dromaeosaurids [23]–[26], and the basal bird *Archaeopteryx* [27]. The promaxillary fenestra is visible in lateral view, as in *Archaeopteryx* [27], dromaeosaurids [23]–[26], the basal troodontid *Sinovenator* [2], and the troodontid *Sinusonasus* [4]. In contrast, the promaxillary fenestra has been lost or has become confluent with the maxillary fenestra in the troodontids *Saurornithoides* and *Zanabazar* [9]. In *Geminiraptor* the promaxillary fenestra is situated at the cranial end of the antorbital fossa. Infilling by matrix prevents determination of its exact dimensions, but the height of the opening is 8 mm or less. The maxillary fenestra of *Geminiraptor* is a large, craniocaudally elongate oval, as in *Troodon*, *Saurornithoides*, and *Zanabazar* [9], [10]. It is 27 mm long and 10.4 mm high. As in most other deinonychosaurs [2], [3], [4], [23]–[26] the fenestra does not reach the cranial margin of the antorbital fossa. In this respect *Geminiraptor* differs from *Troodon*, *Saurornithoides*, and *Zanabazar*, in which the maxillary fenestra reaches the cranial margin of the antorbital fossa [9], [10]. The promaxillary strut, the vertical bar of bone between the promaxillary fenestra and the maxillary fenestra, is craniocaudally narrow in *Geminiraptor*, as in *Sinovenator* [2] and unlike the craniocaudally wide promaxillary strut of *Sinusonasus* [4]. In *Geminiraptor* the interfenestral strut, the vertical bar of bone between the maxillary fenestra and the internal antorbital fenestra, is not recessed inward from the lateral surface of the maxilla. The strut is craniocaudally narrow as in *Sinovenator* [2] and *Sinusonasus* [4] and unlike the craniocaudally wide interfenestral strut in *Byronosaurus*, *Saurornithoides*, and *Zanabazar* [8], [9].

The maxilla of *Geminiraptor suarezarum* is remarkable in that it is inflated medially by a large pneumatic chamber that gives the maxilla a triangular cross-section such that there is a broad, ventrally convex shelf lingual to the tooth row. The bone surrounding this cavity is only ∼2 mm thick. Such extensive pneumatization of this part of the maxilla is unreported in any other theropod except *Byronosaurus*. In *Byronosaurus* the medial wall of the inflated portion is vertical, so a triangular cross-section is absent [8]. In other theropods the maxillary antrum (the space within the inflated area) communicates with the maxillary fenestra [28], although obscuration by matrix prevents confirmation of this in *Geminiraptor*. In *Geminiraptor* the maxillary antrum appears to be closed off from the nasal cavity by a thin medial wall, as is the case in other coelurosaurs [28], including other troodontids [8], [9].

The preserved portion of the maxilla includes alveoli for nine small teeth, dorsal to which is a row of neurovascular foramina on the lateral surface of the maxilla. After comparison with the maxillae of other deinonychosaurs, we estimate that the complete maxilla held at least three more teeth in the missing cranial part and at least seven more in the missing caudal part. The alveoli are square in occlusal view. No tooth crowns or roots are preserved. The alveoli are closely spaced but separated by complete septa. This is significant because in most other troodontids complete septa are absent [10], [29]–[31], although P.S. has observed that they are present in the basal troodontid *Sinovenator changii*, IVPP (Institute of Vertebrate Paleontology and Paleoanthropology, Beijing, China)V 12615. There is no break between the septa and the labial surface of the maxilla and therefore no discrete interdental plates.

### Phylogenetic Analysis

**Figure 4:**
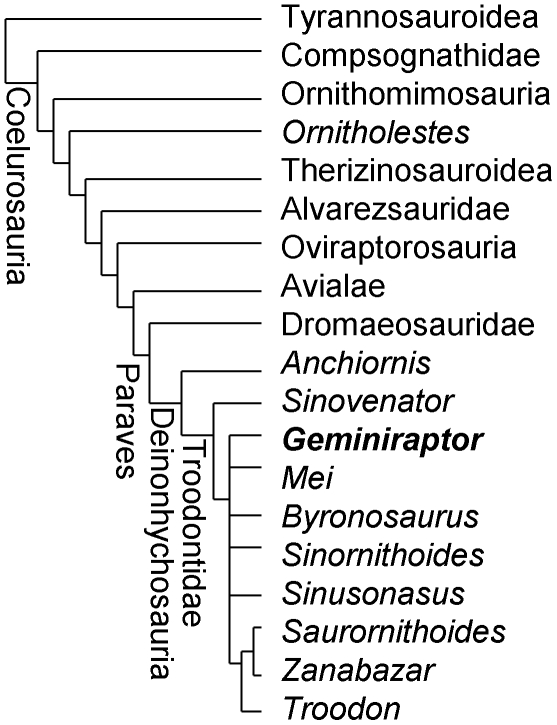
Phylogeny of coelurosaurian theropod dinosaurs, showing phylogenetic position of *Geminiraptor suarezarum*. See text for details of phylogenetic analysis.

The phylogenetic analysis found 1944 trees of 1285 steps. For these trees the consistency index is 0.3416, the homoplasy index is 0.6537, the retention index is 0.7711, and the rescaled consistency index is 0.2670. The strict consensus tree places *G. suarezarum* within Troodontidae in an unresolved polytomy with *Mei*, *Byronosaurus*, *Sinornithoides*, *Sinusonasus*, and the clade *Troodon* + (*Saurornithoides* + *Zanabazar*) (Fig. 4). Successive outgroups to this polytomy are the troodontid *Sinovenator*, the troodontid *Anchiornis*, Dromaeosauridae, and Avialae. Outside Troodontidae the topology of the tree is identical to that found by the latest study that used the present data matrix [13], as is the decay index (Bremer support) at all nodes but one. As in the latest analysis [13], the decay index is a robust 8 for Paraves, a robust 4 for Deinonychosauria, 1 for Troodontidae, and 1 for *Troodon* + (*Saurornithoides* + *Zanabazar*). The decay index dropped from 3 in the previous analysis [13] to 1 in the current analysis, at the node uniting *Sinovenator* with troodontids other than *Anchiornis*. This drop likely reflects the huge amount of missing data for *Geminiraptor*.

*Geminiraptor* is united with troodontids other than *Anchiornis* by presence of a large number of small teeth (character 85, state 1); the plesiomorphic condition, present in *Anchionis* and most other theropods, is a small number of larger teeth (state 0). *Geminiraptor* is united with troodontids other than *Anchiornis* and *Sinovenator* by the presence of a maxillary fenestra in the form of a large, craniocaudally elongate oblong (character 240, state 1); in *Anchiornis*, *Sinovenator*, and most other theropods the maxillary fenestra is large and round (state 0) (Fig. 3). A maxillary fenestra in the form of a large, craniocaudally elongate oblong is unknown outside Troodontidae. Also, the general shape of the maxilla, with its long, low profile, is similar to that of advanced troodontids (Fig. 3), and the bone exhibits no morphology that is inconsistent with troodontid affinity. The presence of interdental septa is shared with *Sinovenator* and non-troodontid theropods and is consistent with a relatively basal position within the family.

## Discussion

The discovery of *G. suarezarum* adds to the known diversity of the dinosaur fauna of the Cedar Mountain Formation. As the only Lower Cretaceous troodontid reported from North America it also increases the known temporal distribution of the family on the continent. It was already known that troodontids were present in Asia and North America during the Late Jurassic [5], [12] and the Late Cretaceous [8]–[10], and we now know that they were present on both continents during the Early Cretaceous as well. This indicates that the family underwent multiple transcontinental dispersal events through its history. However, there are not yet enough data to determine the direction of dispersal or the family's continent of origin

*Geminiraptor* is considerably larger than other Early Cretaceous troodontids [2]–[4], [6] and Jurassic troodontids [5] and is similar in size to the Late Cretaceous troodontids *Byronosaurus* [8], *Troodon* [10], *Saurornithoides* [9], and *Zanabazar* [9]. Its presence in the Early Cretaceous therefore demonstrates that the larger body size characteristic of Late Cretaceous troodontids had already appeared in the Early Cretaceous.

### Nomenclatural Acts

The electronic version of this document does not represent a published work according to the International Code of Zoological Nomenclature (ICZN), and hence the nomenclatural acts contained herein are not available under that code from the electronic edition. A separate edition of this document was produced by a method that assures numerous identical and durable copies, and those copies were simultaneously obtainable (from the publication date listed on page 1 of this article) for the purpose of providing a public and permanent scientific record, in accordance with Article 8.1 of the Code. The separate print-only edition is available on request from PLoS by sending a request to PLoS ONE, 145 Berry Street, Suite 3100, San Francisco, CA 91407, USA along with a check for $10 (to cover printing and postage) payable to “Public Library of Science”.

This published work and the nomenclatural acts is contains have been registered in ZooBank (http://www.zoobank.org), the proposed online registration system for the ICZN. The ZooBank LSIDS (Life Science Identifiers) can be resolved to the associated information viewed through any standard web browser by appending the LSID to the prefix “http://zoobank.org/”.

## Supporting Information

Appendix S1 (0.16 MB DOC)

## References

[1] Makovicky PJ, Norell MA, Weishampel DB, Dodson P, Osmólska H (2004). Troodontidae.. The Dinosauria, Second Edition.

[2] Xu X, Norell MA, Wang X, Makovicky PJ, Wu X (2002). A basal troodontid from the Early Cretaceous of China.. Nature.

[3] Xu X, Norell MA (2004). A new troodontid dinosaur from China with avian-like sleeping posture.. Nature.

[4] Xu X, Wang X (2004). A new troodontid (Theropoda: Troodontidae) from the Lower Cretaceous Yixian Formation of western Liaoning, China.. Acta Geol Sin-Engl.

[5] Hu D, Hou L, Zhang L, Xu X (2009). A pre-*Archaeopteryx* troodontid theropod from China with long feathers on the metatarsus.. Nature.

[6] Russell D, Dong Z (1993). A nearly complete skeleton of a new troodontid dinosaur from the Early Cretaceous of the Ordos Basin, Inner Mongolia, People's Republic of China.. Can J Earth Sci.

[7] Barsbold R, Osmólska H, Kurzanov SM (1987). On a new troodontid (Dinosauria, Theropoda) from the Early Cretaceous of Mongolia.. Acta Palaeontol Pol.

[8] Makovicky PJ, Norell MA, Clark JM, Rowe T (2003). Osteology and relationships of *Byronosaurus jaffei*.. Am Mus Novit.

[9] Norell MA, Makovicky PJ, Bever GS, Balanoff AM, Clark JM, Barsbold R, Rowe T (2009). A review of the Mongolian Cretaceous dinosaur *Saurornithoides*.. Am Mus Novit.

[10] Currie PJ (1985). Cranial anatomy of *Stenonychosaurus inequalis* (Saurischia, Theropoda) and its bearing on the origin of birds.. Can J Earth Sci.

[11] Chure DJ (1994). *Koparion douglassi*, a new dinosaur from the Morrison Formation (Upper Jurassic) of Dinosaur National Monument; the oldest troodontid (Theropoda: Maniraptora).. BYU Geol Stud.

[12] Hartman S, Lovelace D, Wahl W (2005). Phylogenetic assessment of a maniraptoran from the Morrison Formation.. J Vertebr Paleontol.

[13] Senter P (2010). Using creation science to demonstrate evolution: application of a creationist method for visualizing gaps in the fossil record to a phylogenetic study of coelurosaurian dinosaurs.. J Evolution Biol.

[14] Senter P (2007). A new look at the phylogeny of Coelurosauria (Dinosauria: Theropoda).. J Syst Palaeontol.

[15] Swofford DL (2001). PAUP*. Phylogenetic Analysis Using Parsimony (*and Other Methods)..

[16] Owen R (1841). Report on British fossil reptiles, part II.. Report of the eleventh meeting of the British Association for the Advancement of Science.

[17] Seeley (1887). On the classification of the fossil animals commonly named Dinosauria.. P R Soc London.

[18] Marsh OC (1881). Principal characters of American Jurassic dinosaurs.. Part V. Am J Sci.

[19] von Huene F (1914). Das natürliche System der Saurischia.. Centralbl Min Geol Paläontol Abt B.

[20] Gilmore CW (1924). On *Troodon validus*, an ornithopodous dinosaur from the Belly River Cretaceous of Alberta, Canada.. Bull Dept Geol Univ Alb.

[21] Kirkland JI, Madsen SK (2007). The Lower Cretaceous Cedar Mountain Formation, eastern Utah: the view up an always interesting learning curve.. Ut Geol Assoc Publ.

[22] Greenhalgh BW, Britt BB, Willis GC, Hylland MD, Clark DL, Chidsey TC (2007). Stratigraphy and sedimentology of the Morrison-Cedar Mountain boundary, east-central Utah.. Central Utah: diverse geology of a dynamic landscape.

[23] Osborn HF (1924). Three new Theropoda, *Protoceratops* Zone, central Mongolia.. Am Mus Novit.

[24] Ostrom JH (1969). Osteology of *Deinonychus antirrhopus*, an unusual theropod from the Lower Cretaceous of Montana.. Pea Mus Nat Hist Bull.

[25] Xu X, Wu X (2001). Cranial morphology of *Sinornithosaurus millenii* Xu et al. 1999 (Dinosauria: Theropoda: Dromaeosauridae) from the Yixian Formation of Liaoning, China.. Can J Earth Sci.

[26] Makovicky PJ, Apesteguía S, Agnolín FL (2005). The earliest dromaeosaurid theropod from South America.. Nature.

[27] Mayr G, Pohl B, Hartman S, Peters DS (2007). The tenth skeletal specimen of *Archaeopteryx*.. Zool J Linn Soc-Lond.

[28] Witmer LM (1997). The evolution of the antorbital cavity of archosaurs: a study in soft-tissue reconstruction in the fossil record with an analysis of the function of pneumaticity.. Soc Vertebr Paleontol Mem.

[29] Currie PJ (1987). Bird-like characteristics of the jaws and teeth of troodontid theropods (Dinosauria, Saurischia).. J Vertebr Paleontol.

[30] Norell MA, Hwang SH (2004). A troodontid dinosaur from Ukhaa Tolgod (Late Cretaceous, Mongolia).. Am Mus Novit.

[31] Bever GS, Norell MA (2009). The perinate skull of *Byronosaurus* (Troodontidae) with observations on the cranial ontogeny of paravian theropods.. Am Mus Novit.

