# Supplementary material for: A New Troodontid Theropod Dinosaur from the Lower Cretaceous of Utah
Source: PLoS One. 2010 Dec 15;5(12):e14329. doi: 10.1371/journal.pone.0014329 (PMC3002269; doi:10.1371/journal.pone.0014329)
Supplement: Appendix S1 — (0.16 MB DOC) [file pone.0014329.s001.doc]

**Appendix S1: Character List and Matrix for Phylogenetic Analysis**

1. Vaned feathers on forelimb symmetric (0) or asymmetric, with barbs on leading edge of flight feathers shorter than those on trailing edge (1).

2. Orbit round in lateral or dorsolateral view (0) or dorsoventrally elongate (1).

3. Anterior process of postorbital projects into orbit (0) or does not project into orbit (1).

4. Postorbital in lateral view with subhorizontal anterior (frontal) process (0) or frontal process diagonal (anterior tip of process higher than base of process) (1).

5. Postorbital bar parallels quadrate, lower temporal fenestra rectangular in shape (0) or jugal and postorbital approach or contact quadratojugal to constrict lower temporal fenestra (1).

6. Otosphenoidal crest vertical on basisphenoid and prootic, and does not border an enlarged pneumatic recess (0) or well developed, crescent shaped, thin crest forms anterior edge of enlarged pneumatic recess (1). This structure forms the anterior, and most distinct, border of the “lateral depression” of the middle ear region of troodontids and some extant avians.

7. Crista interfenestralis confluent with lateral surface of prootic and opisthotic (0) or distinctly depressed within middle ear opening (1).

8. Subotic recess (pneumatic fossa ventral to fenestra ovalis) absent (0) or present (1)

9. Basisphenoid recess present between basisphenoid and basioccipital (0) or entirely within basisphenoid (1) or absent (2).

10. Posterior opening of basisphenoid recess single (0) or divided into two small, circular foramina by a thin bar of bone (1).

11. Base of cultriform process (parasphenoid rostrum) not highly pneumatized (0) or base of cultriform process expanded and pneumatic (parasphenoid bulla) (1).

12. Basipterygoid processes ventral or anteroventrally projecting (0) or lateroventrally projecting (1).

13. Basipterygoid processes well developed, extending as a distinct process from the base of the basisphenoid (0) or processes abbreviated or absent (1).

14. Basipterygoid processes solid (0) or processes hollow (1).

15. Basipterygoid recesses on dorsolateral surfaces of basipterygoid processes absent (0) or present (1).

16. Depression for pneumatic recess on prootic absent (0) or present as dorsally open fossa on prootic/opisthotic (1) or present as deep, posterolaterally directed concavity (2). The dorsal tympanic recess referred to here is the depression anterodorsal to the middle ear on the opisthotic, not the recess dorsal to the crista interfenestralis within the middle ear as seen in Archaeopteryx lithographica, Shuvuuia deserti and Aves.

17. Accessory tympanic recess dorsal to crista interfenestralis absent (0) small pocket present (1) or extensive with indirect pneumatization (2).

18. Caudal (posterior) tympanic recess absent (0) present as opening on anterior surface of paroccipital process (1) or extends into opisthotic posterodorsal to fenestra ovalis, confluent with this fenestra (2).

19. Exits of C. N. X-XII flush with surface of exoccipital (0) or cranial nerve exits located together in a bowl-like basisphenoid depression (1).

20. Maxillary process of premaxilla contacts nasal to form posterior border of nares (0) or maxillary process reduced so that maxilla participates broadly in external naris (1) or maxillary process of premaxilla extends posteriorly to separate maxilla from nasal posterior to nares (2).

21. Internarial bar rounded (0) or flat (1).

22. Crenulate margin on buccal edge of premaxilla absent (0) or present (1).

23. Caudal margin of naris farther rostral than (0), or nearly reaching or overlapping (1), the rostral border of the antorbital fossa.

24. Premaxillary symphysis acute, V-shaped (0) or rounded, U-shaped (1).

25. Secondary palate short (0) or long, with extensive palatal shelves on maxilla (1).

26. Palatal shelf of maxilla flat (0) or with midline ventral ‘tooth-like’ projection (1)

27. Maxillary fenestra absent (0) or present (1).

28. Maxillary fenestra situated at rostral border of antorbital fossa (0) or situated posterior to rostral border of fossa (1).

29. Promaxillary fenestra absent (0) or present (1).

30. Antorbital fossa without distinct rim ventrally and anteriorly (0) or with distinct rim composed of a thin wall of bone (1).

31. Narial region apneumatic or poorly pneumatized (0) or with extensive pneumatic fossae, especially along posterodorsal rim of fossa (1).

32. Jugal and postorbital contribute equally to postorbital bar (0) or ascending process of jugal reduced and descending process of postorbital ventrally elongate (1).

33. Jugal quadratojugal process tall beneath lower temporal fenestra, and craniocaudally short (0) or rod-like and craniocaudally elongate (1).

34. Jugal pneumatic recess in posteroventral corner of antorbital fossa present (0) or absent (1).

35. Medial jugal foramen present on medial surface ventral to postorbital bar (0) or absent (1).

36. Quadratojugal without horizontal process posterior to ascending process (reversed “L” shape) (0) or with process (i.e., inverted ‘T’ or ‘Y’ shape) (1).

37. Jugal and quadratojugal separate (0) or quadratojugal and jugal fused and not distinguishable from one another (1).

38. Supraorbital crest on lacrimal in adult individuals absent (0) or dorsal crest above orbit (1) or lateral expansion anterior and dorsal to orbit (2).

39. Enlarged foramen or foramina opening laterally at the angle of the lacrimal, absent (0) or present (1).

40. Lacrimal posterodorsal process absent (0) or horizontal and similar in length to the jugal process (1) or horizontal and much shorter than the jugal process or (2) present and vertical.

41. Prefrontal large, dorsal exposure similar to that of lacrimal (0) or greatly reduced in exposure (1) or without exposure (2).

42. Frontals narrow anteriorly as a wedge between nasals (0) or end abruptly anteriorly, suture with nasal transversely oriented (1) or suture with nasals W-shaped (2).

43. Anterior emargination of supratemporal fossa on frontal straight or slightly curved (0) or strongly sinusoidal and reaching onto postorbital process (1).

44. Frontal postorbital process (dorsal view): smooth transition from orbital margin (0) or sharply demarcated from orbital margin (1).

45. Frontal edge smooth in region of lacrimal suture (0) or edge notched (1).

46. Dorsal surface of parietals flat, lateral ridge borders supratemporal fenestra (0) or parietals dorsally convex with very low sagittal crest along midline (1) or dorsally convex with well developed sagittal crest (2).

47. Parietals separate (0) or fused (1).

48. Descending process of squamosal parallels quadrate shaft (0) or nearly perpendicular to quadrate shaft (1).

49. Descending process of squamosal contacts quadratojugal (0) or does not contact quadratojugal (1).

50. Posterolateral shelf on squamosal overhanging quadrate head absent (0) or present (1).

51. Dorsal process of quadrate single headed (0) or with two distinct heads, a lateral one contacting the squamosal and a medial head contacting the braincase (1).

52. Quadrate vertical (0) or strongly inclined anteroventrally so that mandibular end lies far forward of dorsal end (1).

53. Quadrate solid (0) or hollow, with depression on posterior surface (1).

54. Lateral border of quadrate shaft straight (0) or with lateral tab that touches squamosal and quadratojugal above an enlarged quadrate foramen (1)

55. Foramen magnum subcircular, slightly wider than tall (0) or oval, taller than wide (1).

56. Occipital condyle without constricted neck (0) or subspherical with constricted neck (1).

57. Paroccipital process elongate and slender, with dorsal and ventral edges nearly parallel (0) or process short, deep with convex distal end (1).

58. Paroccipital process straight, projects laterally or posterolaterally (0) or distal end curves ventrally, pendant (1).

59. Paroccipital process with straight dorsal edge (0) or with dorsal edge twisted rostrolaterally at distal end (1).

60. Ectopterygoid with constricted opening into fossa (0) or with open ventral fossa in the main body of the element (1).

61. Dorsal recess on ectopterygoid absent (0) or present (1).

62. Flange of pterygoid well developed (0) or reduced in size or absent (1).

63. Palatine and ectopterygoid separated by pterygoid (0) or contact (1).

64. Palatine tetraradiate, with jugal process (0) or palatine triradiate, jugal process absent (1).

65. Suborbital fenestra similar in length to orbit (0) or about half or less than half orbital length (1) or absent (2).

66. Symphyseal region of dentary broad and straight, paralleling lateral margin (0) or medially recurved slightly (1) or strongly recurved medially (2).

67. Dentary symphyseal region in line with main part of buccal edge (0) or abruptly downturned at rostral end (1) or dentary ramus gradually, weakly downturned through its length (2).

68. Surangular without coronoid prominence (0) or with coronoid prominence (1).

69. Posterior end of dentary without posterodorsal process dorsal to mandibular fenestra (0) or with dorsal process above anterior end of mandibular fenestra (1) or with elongate, strongly arched dorsal process extending over most of fenestra (2).

70. Labial face of dentary flat (0) or with lateral ridge and inset tooth row (1).

71. Dorsoventral depth of surangular is (0) or is not greater than that of dentary ramus (1).

72. Nutrient foramina on external surface of dentary superficial (0) or lie within deep groove (1).

73. External mandibular fenestra oval (0) or subdivided by a spinous rostral process of the surangular (1) or external mandibular fenestra absent (2).

74. Internal mandibular fenestra small and slit-like (0) or large and rounded (1).

75. Foramen in lateral surface of surangular rostral to mandibular articulation, absent (0) or present (1).

76. Splenial not widely exposed on lateral surface of mandible (0) or exposed as a broad triangle between dentary and angular on lateral surface of mandible (1).

77. Coronoid ossification large (0) or only a thin splint (1) or absent (2).

78. Articular without elongate, slender medial, posteromedial, or mediodorsal process from retroarticular process (0) or with process (1).

79. Retroarticular process short, stout (0) or elongate and slender (1).

80. Mandibular articulation surface as long as distal end of quadrate (0) or twice or more as long as quadrate surface, allowing anteroposterior movement of mandible (1).

81. Premaxilla toothed (0) or edentulous (1).

82. Second premaxillary tooth approximately equivalent in size to other premaxillary teeth (0) or second tooth markedly larger than third and fourth premaxillary teeth (1) or first premaxillary tooth huge, other premaxillary teeth tiny (2) or first premaxillary tooth larger than the others but all premaxillary teeth tiny (3).

83. Maxilla toothed (0) or edentulous (1).

84. Maxillary and dentary teeth serrated (0) or some without serrations anteriorly (1) or all without serrations (2).

85. Dentary and maxillary teeth large, less than 20 in dentary (0) or large number of small teeth (20 or more in dentary) (1) or small number of dentary teeth (< 11) (2) or dentary without teeth (3).

86. Serration denticles large (0) or small (1).

87. Serrations simple, denticles convex (0) or distal and often mesial edges of teeth with hooked denticles that point toward the tip of the crown (1).

88. Teeth constricted between root and crown (0) or root and crown confluent (1).

89. Dentary teeth evenly spaced (0) or anterior dentary teeth smaller, more numerous, and more closely appressed than those in middle of tooth row (1) or anterior dentary teeth more widely spaced than those in middle of tooth row (2).

90. Dentaries lack distinct interdental plates (0) or with interdental plates medially between teeth (1).

91. In cross section, premaxillary tooth crowns sub-oval to sub-circular (0) or asymmetrical (D-shaped in cross section) with flat lingual surface and both carinae lingually positioned (1) or first premaxillary tooth with flat lingual surface, other premaxillary teeth without flat lingual surfaces (2), or asymmetrical with only the mesial carina lingually offset (3).

92. Number of cervical vertebrae: 10 (0) or 12 or more (1).

93. Axial epipophyses absent or poorly developed, not extending past posterior rim of postzygopophyses (0) or large and posteriorly directed, extend beyond postzygapophyses (1).

94. Axial neural spine flared transversely (0) or compressed mediolaterally (1).

95. Epipophyses of cervical vertebrae placed distally on postzygapophyses, above postzygapophyseal facets (0) or placed proximally, proximal to postzygapophyseal facets (1).

96. Anterior cervical centra level with or shorter than posterior extent of neural arch (0) or centra extending beyond posterior limit of neural arch (1).

97. Carotid process on posterior cervical vertebrae absent (0) or present (1).

98. Anterior cervical centra subcircular or square in anterior view (0) or distinctly wider than high, kidney shaped (1).

99. Cervical neural spines anteroposteriorly long and dorsoventrally tall (0) or anteroposteriorly short, dorsoventrally low, and centered on neural arch, giving arch an “X” shape in dorsal view (1) or anteroposteriorly short and dorsoventrally tall (2) or anteroposteriorly long and dorsoventrally short (3).

100. Cervical centra with one pair of pneumatic openings (0) or with two pairs of pneumatic openings (1).

101. Cervical and anterior trunk vertebrae amphiplatyan (0) or opisthocoelous (1).

102. Anterior trunk vertebrae without prominent hypapophyses (0) or with large hypapophyses (1).

103. Parapophyses of posterior trunk vertebrae flush with neural arch (0) or distinctly projected on pedicels (1).

104. Hyposphene-hypantrum articulations in trunk vertebrae absent (0) or present (1).

105. Zygapophyses of trunk vertebrae abutting one another above neural canal, opposite hyposphenes meet to form lamina (0), or zygapophyses placed lateral to neural canal and separated by groove for interspinous ligaments, hyposphenes separated (1).

106. Middle and posterior dorsal vertebrae not pneumatic (0) or pneumatic (1).

107. Transverse processes of anterior dorsal vertebrae long and thin (0) or short, wide, and only slightly inclined (1).

108. Neural spines of dorsal vertebrae not expanded distally (0) or expanded to form ‘spine table’ (1).

109. Scars for interspinous ligaments terminate at apex of neural spine in dorsal vertebrae (0) or terminate below apex of neural spine (1).

110. Number of sacral vertebrae: 5 (0) or 6 (1) or 7 or more (2).

111. Sacral vertebrae with unfused zygapophyses (0) or with fused zygapophyses forming a sinuous ridge in dorsal view (1).

112. Ventral surface of posterior sacral centra gently rounded, convex (0) or ventrally flattened, sometimes with shallow sulcus (1) or centrum strongly constricted transversely, ventral surface keeled (2).

113. Pleurocoels absent on sacral vertebrae (0) or present on anterior sacrals only (1) or present on all sacrals (2).

114. Last sacral centrum with flat posterior articulation surface (0) or convex articulation surface (1).

115. Caudal vertebrae with distinct transition point (0) or without transition point (1). Here, the transition point is considered the point of abrupt change in vertebral morphology in the tail, at which sudden reduction in the lengths of neural spines and transverse processes occurs, with or without concomitant prezygapophyseal elongation and/or change in hemal arch morphology from dorsoventrally long to dorsoventrally short and shaped like an inverted “T”.

116. Transition point in caudal series begins distal to the 10th caudal (0) or between 7th and 10th caudal vertebra (1) or proximal to the 7th caudal vertebra (2).

117. Anterior caudal centra tall, oval in cross section (0) or with box-like centra in caudals 1 – 5 (1) or anterior caudal centra laterally compressed with ventral keel (2).

118. Neural spines of caudal vertebrae simple, undivided (0) or separated into anterior and posterior alae throughout much of caudal sequence (1).

119. Neural spines on distal caudals form a low ridge (0) or spine absent (1) or midline sulcus in center of neural arch (2).

120. Prezygapophyses of distal caudal vertebrae between 1/3 and whole centrum length (0) or with extremely long extensions of the prezygapophyses (up to 10 vertebral segments long in some taxa) (1) or strongly reduced as in Archaeopteryx lithographica (2).

121. More than 30 caudal vertebrae (0) or 21-30 caudal vertebrae (1) or < 10 caudal vertebrae, followed by pygostyle (2) or 11 – 20 vertebrae (3).

122. Proximal end of chevrons of proximal caudals short anteroposteriorly, shaft proximodistally elongate (0) or proximal end elongate anteroposteriorly, flattened and plate-like (1).

123. Distal caudal chevrons are simple (0) or anteriorly bifurcate (1) or bifurcate at both ends (2).

124. Shaft of cervical ribs slender and longer than vertebra to which they articulate (0) or broad and shorter than vertebra (1).

125. Ossified uncinate processes absent (0) or present (1).

126. Ossified ventral rib segments absent (0) or present (1).

127. Lateral gastralial segment shorter than medial one in each arch (0) or distal segment longer than proximal segment (1).

128. Ossified sternal plates separate in adults (0) or fused (1).

129. Sternum without distinct lateral xiphoid process posterior to costal margin (0) or with lateral xiphoid process (1).

130. Anterior edge of sternum grooved for reception of coracoids (0) or sternum without grooves (1).

131. Articular facet of coracoid on sternum: anterolateral or more lateral than anterior (0); almost anterior (1).

132. Hypocleidium on furcula absent or very small (0) or a prominent prong (1).

133. Acromion margin of scapula continuous with blade (0) or anterior edge laterally everted (1).

134. Anterior surface of coracoid ventral to glenoid fossa unexpanded (0) or anterior edge of coracoid expanded, forms triangular subglenoid fossa bounded laterally by coracoid tuber (1).

135. Scapula and coracoid separate (0) or fused into scapulocoracoid (1).

136. Coracoid in lateral view subcircular, with shallow ventral blade (0) or subquadrangular with extensive ventral blade (1) or shallow ventral blade with elongate posteroventral process (2) or subtriangular (proximal end constricted, distal end wide) (3).

137. Scapula and coracoid form a continuous arc in posterior and anterior views (0) or coracoid inflected medially, scapulocoracoid ‘L’ shaped when viewed along edge of scapular blade (1).

138. Glenoid fossa without (0) or with extension of glenoid floor onto external surface of scapula (the surface opposite the costal surface) (1).

139. Scapula longer than humerus (0) or humerus longer than scapula (1).

140. Deltopectoral crest large and distinct, proximal end of humerus quadrangular in anterior view (0) or deltopectoral crest less pronounced, forming an arc rather than being quadrangular (1) or deltopectoral crest very weakly developed, proximal end of humerus with rounded edges (2) or deltopectoral crest extremely long (3) or proximal end of humerus extremely broad, triangular in anterior view (4).

141. Anterior surface of deltopectoral crest smooth (0) or with distinct groove or ridge near lateral edge along distal end of crest (1).

142. Olecranon process weakly developed (0) or distinct and large but not hypertrophied (1) or hypertrophied (2).

143. Distal articular surface of ulna flat (0) or convex, semilunate surface (1).

144. Proximal surface of ulna a single continuous articular facet (0) or divided into two distinct fossae separated by a median ridge (1).

145. Lateral proximal carpal (ulnare?) quadrangular (0) or triangular in proximal view (1).

146. Two distal carpals in contact with metacarpals, one covering the base of metacarpal I (and perhaps contacting metacarpal II) the other covering the base of metacarpal II (distal carpals 1 and 2 unfused) (0) or a single distal carpal capping metacarpals I and II (distal carpals 1 and 2 fused) (1).

147. Distal carpals not fused to metacarpals (0) or fused to metacarpals, forming carpometacarpus (1).

148. Distal carpals 1+2 well developed, covering all of proximal ends of metacarpals I and II (0) or small, cover about half of base of metacarpals I and II (1) or cover bases of all metacarpals (2).

149. Metacarpal I half or less than half the length of metacarpal II, and longer proximodistally than wide transversely (0) or subequal in length to metacarpal II (1) or very short and wider transversely than long proximodistally (2) or about two-thirds the length of metacarpal II (3).

150. Third manual digit present, phalanges present (0) or reduced to no more than metacarpal splint (1).

151. Flexor tubercles of manual unguals proximal (0) or displaced distally from articular end (1) or proximodistally elongated with proximal end close to articular facet (2).

152. Unguals on first two digits generally similar in size (0) or digit I bearing large ungual and unguals of other digits distinctly smaller (1).

153. Pronounced proximodorsal ‘lip’ (transverse ridge immediately dorsal to the articulating surface) on first manual ungual absent (0) or present (1).

154. Ventral edge of anterior ala of ilium straight or gently curved (0) or ventral edge hooked anteriorly (1).

155. Preacetabular part of ilium roughly as long as postacetabular part of ilium (0) or preacetabular portion of ilium markedly longer (more than 2/3 of total ilium length) than postacetabular part (1).

156. Anterior end of ilium gently rounded or straight (0) or anterior end strongly curved (1) or pointed at anterodorsal corner (2) or with notch at anterodorsal corner (3).

157. Supraacetabular crest on ilium as a separate process from antitrochanter, forms “hood” over femoral head present (0) reduced, not forming hood (1) or absent (2).

158. Postacetabular ala of ilium in lateral view squared (0) or sharply acuminate (1).

159. Postacetabular blades of ilia in dorsal view parallel (0) or diverge posteriorly (1).

160. Tuber along dorsal edge of ilium, dorsal or slightly posterior to acetabulum absent (0) or present (1).

161. Brevis fossa shelf-like (0) or deeply concave with lateral overhang (1).

162. Antitrochanter posterior to acetabulum absent or poorly developed (0) or prominent (1).

163. Ridge bordering cuppedicus fossa extends far posteriorly and is confluent or almost confluent with acetabular rim (0) or ridge terminates rostral to acetabulum or curves ventrally onto anterior end of pubic peduncle (1).

164. Cuppedicus fossa deep, ventrally concave (0) or fossa shallow or flat, with no lateral overhang (1) or absent (2).

165. Posterior edge of ischium without (0) or with prominent proximodorsal prong (1).

166. Shaft of ischium straight in lateral view (0) or ventrodistal end curved anteriorly (1) or curved dorsally (posterodorsally concave) (2).

167. Obturator process of ischium absent (0) or proximal in position (1) or distally displaced (2).

168. Obturator process does not contact pubis (0) or contacts pubis (1).

169. Length of pubic boot < 30% length of pubis (0) or > 40% (1).

170. Semicircular scar on posterior part of the proximal end of the ischium, absent (0) or present (1).

171. Ischium more than 70% (0) or 70% or less of pubis length (1).

172. Distal ends of ischia form symphysis (0) or approach one another but do not form symphysis (1) or widely separated (2). .

173. Ischial boot (expanded distal end) present (0) or absent (1).

174. Length of pubis not reduced (similar to that of femur) (0) or strongly reduced (1).

175. Pubis propubic (0) or pubis vertical (1) or pubis moderately posteriorly oriented (2) or pubis fully posteriorly oriented (opisthopubic) (3). The oviraptorid condition, in which the proximal end of the pubis is vertical and the distal end curves anteriorly, is considered to be state 1.

176. Pubic boot projects anteriorly and posteriorly (0) or with little or no anterior process (1) or no anteroposterior projections (2).

177. Shelf on pubic shaft proximal to symphysis (‘pubic apron’) extends medially from middle of cylindrical pubic shaft (0) or shelf extends medially from anterior edge of anteroposteriorly flattened shaft (1).

178. Pubic shaft straight (0) or distal end curves anteriorly, anterior surface of shaft concave in lateral view (1) or anterior surface of shaft convex in lateral view (2).

179. Pubic apron about half of pubic shaft length (0) or less than 1/3 of shaft length (1).

180. Femoral head without fovea capitis (for attachment of capital ligament) (0) or circular fovea present in center of medial surface of head (1).

181. Lesser and greater trochanters unfused (0) or fused (1).

182. Lesser trochanter of femur alariform (0) or cylindrical in cross section (1).

183. Posterior trochanter absent or represented only by rugose area (0) or posterior trochanter distinctly raised from shaft, mound-like (1).

184. Fourth trochanter on femur present (0) or absent (1).

185. Accessory trochanteric crest distal to lesser trochanter absent (0) or present (1).

186. Anterior surface of femur proximal to medial distal condyle without longitudinal crest (0) or crest present extending proximally from medial condyle on anterior surface of shaft (1).

187. Popliteal fossa on distal end of femur open distally (0) or closed off distally by contact between distal condyles (1).

188. Fibula reaches proximal tarsals (0) or short, tapering distally, and not in contact with proximal tarsals (1).

189. Medial surface of proximal end of fibula concave along long axis (0) or flat (1).

190. Deep oval fossa on medial surface of fibula near proximal end absent (0) or present (1).

191. Distal end of tibia and astragalus without distinct condyles (0) or with distinct condyles separated by prominent tendinal groove on anterior surface (1).

192. Medial cnemial crest absent (0) or present on proximal end of tibia (1).

193. Ascending process of the astragalus tall and broad, covering most of anterior surface of distal end of tibia (0) or process short and slender, covering only lateral half of anterior surface of tibia (1) or ascending process tall with medial notch that restricts it to lateral side of anterior face of distal tibia (2).

194. Ascending process of astragalus confluent with condylar portion (0) or separated by transverse groove or fossa across base (1).

195. Astragalus and calcaneum separate from tibia (0) or fused to each other and to the tibia in late ontogeny (1).

196. Distal tarsals separate, not fused to metatarsals (0) or form metatarsal cap with intercondylar prominence that fuses to metatarsal early in postnatal ontogeny (1).

197. Metatarsals not co-ossified (0) or co-ossification of metatarsals begins proximally (1) or distally (2).

198. Distal end of metatarsal II smooth, not ginglymoid (0) or with developed ginglymus (1).

199. Distal end of metatarsal III smooth, not ginglymoid (0) or with developed ginglymus (1).

200. In anterior view, metatarsal III not pinched (0) or pinched proximally (1) or pinched both proximally and through midshaft (2).

201. Ungual of pedal digit II similar in size to that of III (0) or pedal ungual II about 50% larger than pedal ungual III (1).

202. Metatarsal I articulates at middle of metatarsal II (0) or metatarsal I attaches to distal quarter of metatarsal II (1) or metatarsal I articulates with metatarsal II near its proximal end (2) or metatarsal I absent (3).

203. Metatarsal I attenuates proximally (0) or proximal end of metatarsal I similar to that of metatarsals II-IV (1).

204. Shaft of MT IV round or thicker dorsoventrally than wide in cross section (0) or shaft of MT IV mediolaterally widened and flat in cross section (1).

205. Foot symmetrical (0) or asymmetrical with slender MTII and very robust MT IV (1).

206. Neural spines on posterior dorsal vertebrae in lateral view rectangular or square (0) or anteroposteriorly expanded distally, fan-shaped (1).

207. Shaft diameter of manual phalanx I-1 less (0) or greater (1) than shaft diameter of radius.

208. Angular exposed almost to end of mandible in lateral view, reaches or almost reaches articular (0) or excluded from posterior end angular suture turns ventrally and meets ventral border of mandible rostral to glenoid (1).

209. Laterally inclined flange along dorsal edge of surangular for articulation with lateral process of lateral quadrate condyle absent (0) or present (1).

210. Distal articular ends of metacarpals I + II ginglymoid (0) or rounded, smooth (1).

211. Radius and ulna well separated (0) or with distinct adherence or syndesmosis distally (1).

212. Kink and downward deflection in dentary buccal margin at rostral end of dentary: absent (0) or present (1).

213. Quadrate head covered by squamosal in lateral view (0) or quadrate cotyle of squamosal open laterally exposing quadrate head (1).

214. Brevis fossa poorly developed adjacent to ischial peduncle and without lateral overhang, medial edge of brevis fossa visible in lateral view (0), or fossa well developed along full length of postacetabular blade, lateral overhang extends along full length of fossa, medial edge completely covered in lateral view (1).

215. Vertical ridge on lesser trochanter present (0) or absent (1).

216. Supratemporal fenestra bounded laterally and posteriorly by the squamosal (0) or supratemporal fenestra extended as a fossa on to the dorsal surface of the squamosal (1).

217. Dentary fully toothed (0) or only with teeth rostrally (1).

218. Posterior edge of coracoid not or shallowly indented below glenoid (0), or posterior edge of coracoid deeply notched just ventral to glenoid, glenoid lip everted (1).

219. Retroarticular process points caudally (0) or curves gently dorsocaudally (1).

220. Flange on supraglenoid buttress on scapula absent (0) or present (1).

221. Depression (possibly pneumatic) on ventral surface of postorbital process of laterosphenoid absent (0) or present (1).

222. Basal tubera set far apart, level with or beyond lateral edge of occipital condyle and/or foramen magnum (may connected by a web of bone or separated by a large notch) (0) or tubera small, directly below condyle and foramen magnum, and separated by a narrow notch (1).

223. Basioccipital without pneumatization on occipital surface (0) or with subcondylar recess (1).

224. Ventral surface of dentary straight or nearly straight (0) or descends strongly posteriorly (1).

225. Distal humerus with small or no medial epicondyle (0) or with large medial epicondyle, medial condyle centered on distal end (1).

226. Distal humeral condyles on distal end (0) or on anterior surface (1).

227. Ilium and ischium articulation flat or slightly concavo-convex (0) or ilium with process projecting into socket in ischium (1).

228. Roots of dentary and maxillary teeth mediolaterally compressed (0) or circular in cross-section (1).

229. Preacetabular portion of ilium parasagittal (0) moderately laterally flaring (1) strongly laterally flaring (2).

230. Maxillary and dentary teeth labiolingually flattened and recurved, with crowns in middle of tooth row more than twice as high as the basal mesiolateral width (0) or lanceolate and subsymmetrical (1) or conical (2) or labiolingually flattened and recurved, with crowns in middle of tooth row less than twice as high as the basal mesiolateral width (3).

231. Dentary teeth do not (0) or do increase in size anteriorly, becoming more conical in shape (1).

232. In adults, length of skull more than 90% femoral length (0) or less than 80% (1).

233. Height of skull (minus mandible) at middle of naris more than half the height of skull at middle of orbit (0) or less than half (1).

234. Dorsal margin of naris below level of dorsal margin of orbit (0) or above (1).

235. In lateral view, snout does not (0) or does taper to an anterior point (1).

236. Area of antorbital fenestra greater than that of orbit (0) or less than that of orbit (1).

237. Length of external naris similar to that of orbit (0) or much shorter than orbit (1).

238. Antorbital fossa anteriorly bounded by maxilla (0) or by premaxilla (1).

239. Maxillary antorbital fossa: small, from 10% to less than 40% of the rostrocaudal length of the antorbital cavity (0), large, greater than 40% of the rostrocaudal length of the antorbital cavity (1).

240. Maxillary fenestra large and round (0), a large, craniocaudally elongate oblong (1), a small, craniocaudally elongate slit, not dorsally displaced (2), or a dorsally displaced opening (3).

241. Nasal fusion: absent, nasals separate (0), present, nasals fused together (1).

242. Nasal surface: smooth (0), rugose (1).

243. Suborbital process of jugal short and dorsoventrally stout (0) or elongate and dorsoventrally narrow (1).

244. Nasals at least as long as frontals (0) or shorter than frontals (1).

245. Cross-section of pedal unguals III and IV triangular (0) or a vertical oval (1).

246. Jugo-maxillary bar at ventral end of antorbital fenestra dorsoventrally deep (0) or dorsoventrally narrow (1).

247. Anteroventral corner of premaxilla does not (0) or does form an acute, ventrally oriented point in lateral view (1).

248. Length of preorbital region of cranium > height at anterior edge of preorbital bar (exclusive of midline sagittal ridge, if any) (0) or < height at anterior edge of preorbital bar (1).

249. Frontals without supraorbital rim (0) or with supraorbital rim (1).

250. Parietals shorter than frontals (0) or longer (1).

251. Length of ventral border of infratemporal fenestra comparable to that of orbit (0) or much shorter (1).

252. Foramen magnum smaller than or subequal to size of occipital condyle (0) or larger than occipital condyle (1).

253. Dentary not bowed (0) or bowed (concave dorsally) (1).

254. Meckelian groove of dentary deep (0) or shallow (1).

255. Dentary without posteroventral process extending to posterior end of external mandibular fenestra (0) or with such a process (1).

256. Horizontal shelf on the lateral surface of the surangular, rostral and ventral to the mandibular condyle: absent or faint ridge (0), prominent and extending laterally (1).

257. Premaxillary teeth subequal in size to (0) or much smaller than (1) the maxillary teeth.

258. Approximately the same number of denticles per 5 mm on mesial keels of teeth as on distal keels (0) or markedly more denticles per 5 mm on mesial keels (1).

259. Lateral trochanter (new term for lateral flange on proximal end of femur as in *Caudipteryx* and *Microvenator*) absent (0) or present (1).

260. Dentary tooth implantation: in sockets (0), in paradental groove (1).

261. Dentary dentition continues cranially to tip of dentary (0) or terminates before reaching dentary tip (1).

262. Length of mid-cervical centra approximately the same as dorsal centra (0) or markedly longer than dorsal centra (1).

263. Cervical prezygapophyses unflexed (0) or flexed (1).

264. Dorsal centra > 1.2 x taller than long (0) or height < length (1).

265. Posterior dorsal neural spines > 1.5 x taller than long (0) or height < 1.5 x length (1).

266. Postzygapophyses of middle and posterior dorsal vertebrae do not extend posterior to centrum (0) or do (1).

267. Anteriormost hemal arches > 1.5 x longer than associated centra (0) or < 1.5 x as long as centra (1).

268. Angle between furcular arms > 80^o^ (0) or < 60^o^ (1).

269. Acromion process contacts coracoid (0), or reduced and does not contact coracoid (1).

270. Acromion process does not match any of the following descriptions: (0) rectangular with its dorsal edge forming a 90^o^ angle with the dorsal edge of the scapular blade (1) or a quarter-circle in shape (2) or triangular, with apex pointing away from and subparallel to scapular blade (3).

271. Scapulocoracoid dorsal margin: pronounced notch between the acromion process and the coracoid (0) or margin smooth (1).

272. Wide distal expansion of scapula absent (0) or present (1).

273. Acrocoracoid process absent (0) or present (1).

274. Humeral length is half femoral length or less (0) or shorter than femur but more than half femoral length (1) or longer than femur (2).

275. Length of humeral shaft between deltopectoral crest and distal condyles < 4.5 x shaft diameter (0) or > 4.5 x shaft diameter (1).

276. UIna not bowed away from radius (0), or bowed away from radius (1).

277. Length of radius < 1/3 femoral length (0) or between 1/3 and 2/3 femoral length (1) or between 2/3 and 1x femoral length (2) or > femoral length (3).

278. Radial diameter > 0.5 x ulnar diameter (0) or < 0.5 x (1).

279. Distal carpals 1+2 flattish (0) or semilunate in shape (1).

280. Length of manual digit II (including metacarpal) less than 1.25 x femoral length (0) or > 1.25 x femoral length (1).

281. Distal end of metacarpal I medially (0) or laterally rotated (1).

282. Medial side of metacarpal II: expanded proximally (0), not expanded (1).

283. Metacarpal III > 0.75 x length of metacarpal II (0) or < 0.75 x (1).

284. Manual phalanx I-1 longer than metacarpal II (0) or shorter or subequal in length (1).

285. Length of metacarpal II < length of metacarpal I + phalanx I-1 (0) or > (1).

286. Metacarpals II and III are not (0) or are appressed for their entire lengths (1).

287. Proximal end of metacarpal III is not (0) or is mainly palmar to that of metacarpal II (1).

288. Length of manual phalanx II-2 < 1.2 x length of phalanx II-1 (0) or > 1.2 x (1).

289. Medial ligament pits of manual phalanges deep (0) or shallow (1).

290. Posterior flange on manual phalanx II-1 absent (0) or present (1).

291. Combined lengths of manual phalanges II-1 and II-2 > length of metacarpal II +carpus (0) or < length of metacarpal II +carpus (1).

292. Length of manual phalanx II-1 < 2 x length of III-1 (0) or > 2 x length of III-1 (1).

293. Length of manual phalanx II-2 < 2 x length of II-1 (0) or > 2 x (1).

294. Length of manual phalanx III-1 < 2 x length of phalanx III-2 (0) or > 2 x (1).

295. Manual phalanx I-1 straight (0) or bowed (palmar surface concave) (1).

296. With proximal articular surface of ungual oriented vertically, dorsal surface of manual ungual I does not (0) or does arch higher than level of dorsal extremity of proximal articular surface (1).

297. With proximal articular surface of ungual oriented vertically, dorsal surface of manual ungual II does not (0) or does arch higher than level of dorsal extremity of proximal articular surface.

298. Manual ungual I strongly curved (0), weakly curved (1), or straight (2).

299. Manual unguals II and III strongly curved (0), weakly curved, (1), or straight (2).

300. Pronounced proximodorsal “lip” on manual unguals II and III absent (0) or present (1).

301. Manual digit III with four phalanges (0) or less than four phalanges (1).

302. Manual phalanx III-3 markedly shorter than combined lengths of phalanges III-1 and III-2 (0), subequal in length to their combined lengths (1), or markedly longer (2).

303. Arching of preacetabular iliac blade above height of postacetabular blade absent or small (0) or extreme (1).

304. Shaft of ischium: subequal in thickness to the pubis (0), slenderer than the pubic shaft (1), thicker than the pubic shaft (2).

305. Obturator process does not (0) or does form a strongly acute angle in lateral view (1).

306. Obturator process does not (0) or does reach tip of ischium (1).

307. Ventral notch between the distal portion of the obturator process and the shaft of the ischium: present (0), absent (1).

308. Strong kink of pubis at midshaft absent (0) or present, displacing distal half of pubis caudally (1).

309. In adult, femur longer than tibia (0) or shorter (1).

310. Tip of lesser trochanter below level of femoral head (0) or level with femoral head (1).

311. Proximolateral (fibular) condyle of the tibia, development in proximal view: bulge from the main surface of the tibia (0), conspicuous narrowing between the body of the condyle and the main body of the tibia (1).

312. Metatarsus less than half length of femur (0) or more than half femoral length (1).

313. Metatarsal cross-sectional proportions: subequal or wider mediolaterally than craniocaudally at midshaft (0), deeper craniocaudally than mediolaterally at midshaft (1).

314. Shafts of metatarsals not appressed (0) or appressed (1).

315. Length of metatarsal V > 0.5 x length of metatarsal IV (0) or < 0.5 x (1).

316. Marked mediolateral decrease in transverse width of metatarsus distally, absent (0) or present (1).

317. Plantar surface of hallux faces posteriorly (0) or hallux reoriented so that plantar surface faces medially or anteriorly (1).

318. Hallucal ungual reduced in size relative to other pedal unguals (0) or not reduced (1).

319. Hallucal ungual weakly curved (0) or strongly curved (1).

320. Length of pedal phalanx II-2 between 0.6 x and 1 x length of phalanx II-1 (0), < 0.6 x, or (1) > 1 x (2).

321. Total length of pedal phalanx II-2 (not counting posteroventral lip, if any) > 2 x length of distal condylar eminence (0) or < 2 x (1).

322. Pedal phalanx II-2 without posteroventral lip or keel (0) with transversely wide posteroventral lip (1) with transversely narrow posteroventral keel (2).

323. Pedal phalanx II-1 without dorsal extension of distal condyles (0) or with extension (1).

324. Pedal unguals III and IV straight or weakly curved (0), or strongly curved (1).

325. With fingers extended, tip of ungual III extends no further distally than flexor tubercle of ungual II (0) or extends further (1).

326. Manual ungual III smaller than ungual II (0) or approximately the same size (1).

327. Diameter of non-ungual phalanges of manual digit III > 0.5 x diameter of non-ungual phalanges of digit II (0) or < 0.5 x (1).

328. Manual phalanx II-1 shorter than I-1 (0) or longer (1).

329. Ischial shaft rodlike (0) or flat, platelike (1).

330. Lateral face of ischial shaft flat (or round in rodlike ischia) (0) or laterally concave (1) or with longitudinal ridge dividing lateral surface into anterior and posterior parts (2).

331. Contact between pubic apron contributions of both pubes meet extensively (0) or contact interrupted by a slit (1) or no contact (2).

332. Dorsal margin of postacetabular iliac blade straight or convex (0) or concave (1).

333. Large, longitudinal flange along caudal or lateral face of metatarsal IV absent (0) or present (1).

334. Distally placed dorsal process along caudal edge of ischial shaft absent (0) or present (1).

335. Length of metatarsus < 3.5x transverse midshaft diameter (0) or 3.5x – 8x midshaft diameter (1) or > 8x midshaft diameter (2).

336. Lengths of mid-caudal centra subequal to or less than those of proximal caudal centra (0) or > twice as long as proximal caudal centra (1).

337. Pubic peduncle of ilium craniocaudally longer (0) or shorter (1) than ischial peduncle of ilium.

338. Phalanges of pedal digit III not blocky (proximal phalanx length > 2x diameter) (0) or blocky (proximal phalanx length < 2x diameter) (1).

339. Width of distal humeral expansion < 1/3 humeral length (0) or > 1/3 humeral length (1).

340. Lateral epicondyle of humerus not expanded laterally (0) or expanded laterally (1).

341. Distal end of metatarsal I reduced in size relative to distal ends of other metatarsals (0) or comparable in size to distal ends of other metatarsals (1).

342. Pedal phalanx II-1 longer (0) or shorter (1) than pedal phalanx IV-1.

343. Dentary ramus elongate (0) or shortened, not much longer than tall (1).

344. Metacarpal II > 1/3 humeral length (0) or < 1/3 humeral length (1).

345. With fingers extended, tip of ungual I does not extend past flexor tubercle of ungual II (0) or extends past flexor tubercle of ungual II but does not extend past tip of ungual II (1) or extends past tip of ungual II (2).

346. Premaxillary teeth serrated (0) or unserrated (1).

347. Sublacrimal process of jugal dorsoventrally expanded (taller than suborbital bar of jugal) (0) or not dorsoventrally expanded (1).

348. Flexor tubercles of manual unguals > 1/3 x height of articular facet (0) or < 1/3 (1).

349. Distal chevrons straight or L-shaped in lateral view (0) or upside-down T-shaped (1).

350. Metacarpal III distally not ginglymoid (0) or ginglymoid (1).

351. Breadth of acromion process perpendicular to long axis of scapular blade: deep (0) or shallow (1).

352. Proximal end of metatarsal IV curls around plantar side of proximal end of metatarsal III (0) or doesn’t (1).

353. Midsagittal ridge formed by dorsal displacement of midline of frontals, nasals, and premaxillae absent (0) or present (1).

354. Ectopterygoid lateral to pterygoid (0) or rostral to pterygoid (1).

355. Palatine-pterygoid-ectopterygoid bar does not (0) or does (1) arch below ventral cheek margin.

356. Coossification of angular and surangular absent (0) or present (1).

357. Cervical ribs unfused to cervical vertebrae (0) or fused to cervical vertebrae (1).

358. Anteroproximal contact between metatarsals II and IV absent (0) or present (1).

359. Anterior caudal vertebrae without pneumatopores (0) or with pneumatopores (1).

360. External mandibular fenestra not rostrally displaced (sits beneath orbit) (0) or rostrally displaced (sits anterior to orbit) (1).

361. Distinct notch demarcating proximal end of flexor tubercle of pedal ungual II in lateral view absent (0) or present (1).

362. Ossified sternum demonstrably absent (0) or present (1)

363. Length of manual unguals distal to flexor tubercle is much greater than height of articular facet (0) or is not (1)

364. Difference in lengths of toes II and IV (exclusive of unguals) small (0) or extreme, with toe II shorter (1)

In the *Geminiraptor* line, data in bold font represent characters for which the description refers to the dentary, but the character state in the dentary correlates with a specific state in the maxilla (for example, if the dentary lacks interdental plates, so does the maxilla, so a character referring to presence/absence of discrete interdental plates in the dentary can be scored for the maxilla).

????? ????? ????? ????? ????1 01110 ????? ????? ????? ?????

????? ????? ????? ????? ????? ????? ??0?**1** ????**0** ????? ?????

????? ????? ????? ????? ????? ????? ????? ????? ????? ?????

????? ????? ????? ????? ????? ????? ????? ????? ????? ?????

????? ????? ????? ????? ????? ????? ????? ??011 ????? ?????

????? ????**0** ????? ????? ????? ????? ????? ????? ????? ?????

????? ????? ????? ????? ????? ????? ????? ????? ????? ?????

????? ????? ????

*Dilophosaurus wetherilli*

?1000 00000 00000 00010 00000 00?00 0001? 00102 ????? ??010

00000 0000? ????? 00000 00000 00000 00000 00100 00100 00030

10110 00000 00001 ?0000 00000 0???? ??000 00000 0100? 10100

01000 00000 00000 00000 00000 20000 00000 00000 00000 00000

00000 00000 00000 ?0000 00000 00000 00000 0000? ?0000 0000?

00000 10000 00010 00?00 01000 01000 00010 00000 00000 00000

00000 00000 01000 000?0 00001 00000 00000 00000 00000 00000

000?? 00000 0000

*Allosaurus fragilis*

?1100 0?000 00000 00010 00100 01000 00011 00112 0201? 20000

00000 00001 00000 00000 00001 00000 00000 10101 00100 00000

10010 00000 00001 ?0100 00000 01??? ?0000 00000 01000 10100

01010 00000 10000 01010 00000 00000 00001 10000 00100 00000

00000 00000 00100 00000 00000 00000 00000 00000 00000 00000

00000 10000 00000 00000 00000 00000 00000 00000 01000 00000

00000 00000 00000 00000 00000 01000 00000 00000 00000 00000

00000 00000 0000

*Sinraptor dongi*

?1100 0??00 ?0000 00010 00100 010?0 00000 00112 02010 2?000

00000 00001 0000? 00000 000?1 0?000 00000 10101 00100 00000

10010 00000 00001 ?0??? ????? ????? 0?0?0 ??00? ????? ????0

0??10 000?0 100?0 01000 00000 00000 00001 10000 00100 00000

00000 0?100 ?000? 00?00 0000? ?0000 00000 00000 00000 00000

00000 10000 00010 0??00 ?0??? ????0 ?00?? 00?0? ????? ??00?

??020 00000 00001 0?000 0000? ?1?00 00000 ?00?? 000?? 001?0

00000 00000 0?00

*Dilong paradoxus*

??100 ????? 000?? ????? 0011? ?1010 0000? 0?012 1?0?? 2?000

??1?? 0?0?? ????? 000?0 00??0 ???00 00000 1010? 1???0 0?010

1???? 0???? ????1 ????0 ?0?0? 1?0?? ????0 00?10 ????? ?0?00

00000 ??0?? ????0 ?101? 0?00? 1?0?? ?0??? ??0?? ????? ???10

0?0?? 00??0 ?0??? ?0?0? ???0? ??0?0 00000 00003 1000? 00000

0?0?? ?110? 00011 00?00 01011 ?1??0 0?100 00100 01000 00000

01?00 01010 ?1011 00000 00000 010?? ?0?01 ?00?? 0?0?0 00010

0?0?? ?0000 ??00

*Guanlong wucaiwanensis*

??1?0 ???0? ????? ????0 0011? ?1110 1000? 000?2 1?0?? ??000

?0??0 ?000? ????? ?0000 ?00?0 0?000 00000 1?10? 10??? ?0???

?0??? 0?0?? ?000? ????0 ???10 0???? ????0 0??00 ?1??? 10100

00010 30000 10000 0101? 0?001 0?0?? 00001 ?00?? 0???0 00??0

000?0 00100 ?0?0? 00?0? ?0100 01?00 00000 00000 10000 00000

?00?0 0110? 00?11 0??0? ?1011 11010 00010 001?0 01000 00000

0100? 00010 ?1?11 0???0 000?0 00000 ?0001 ?0000 00010 000??

0?0?? 00?00 0?00

*Eotyrannus lengi*

????? ????? ????? ????? ?0?1? ????? ????? ??0?? ????? ?????

????? ????? ????? 00??0 ?0??? ????? 0000? 10??? 1???0 0??1?

1???? ????? ??1?? ????? ????? ????? ??0?0 ?0?00 0???? ????0

00??? ????? ????? ????? ????? ????? ????? ????? ?0??? 00???

????? ????0 ?0??? ?00?? ????0 0???0 0??00 ?00?? 11??? ?0???

??01? 111?0 0?0?? ???00 ????1 1?0?? 0??1? 0?0?? 0?0?? 0?00?

????? ????? ????? ????? ????? ??1?? ????1 ??000 ??0?0 0?0??

?00?? ????? ??0?

*Raptorex kriegsteini*

?11?? ????? 0???? ????? ?001? 01010 000?? 00012 1201? 2????

????? ????? ????? 00000 00??1 0??00 00000 1?00? 101?0 0??1?

10??? 1???0 ??1?? ????? ???0? ????? ??000 00001 010?? ?0???

??010 210?0 1???0 01??? ???0? 0?0?? 00?01 0???? 0?010 ???01

????? 00100 ?0?0? ?0000 ???00 0??00 00000 0?000 1100? ?000?

??0?? 11?0? 00010 0??01 01001 000?? 0???? ??1?0 ??0?0 0?0??

??01? 01011 ?1??? ??00? ????? ??000 ?0?0? 00?00 ??0?? 000??

0?0?? 00?00 ??0?

*Tyrannosaurus rex*

?1000 0?00? 00000 00210 00011 01010 00000 00012 12010 21100

00100 10001 ?0000 00000 00001 00100 00000 10101 10100 00000

10010 10000 00101 ?0000 0000? ?1??? ?0000 00001 0100? 00101

00010 31000 10000 01011 01101 00000 00001 00001 00010 00002

00000 00100 00000 00000 00100 01000 00000 00010 11000 00000

00010 11000 00000 00001 01000 00000 01110 00100 0?0?0 0?0??

??010 01001 01011 0?000 0000? ??000 10000 00000 00010 00010

00000 00100 0?00

*Gorgosaurus libratus*

?1000 0?00? 000?0 ???10 00011 01110 00000 00112 1201? 21100

?0100 10001 ????0 00000 00001 00100 00000 10101 10100 0000?

?0010 10000 00101 ?0000 00000 01??? ?0000 00001 01000 00101

00010 31000 10000 01011 00101 00000 00001 00001 00010 00002

00000 00100 00000 00000 ?0100 01000 00000 00010 11000 00100

00010 11000 00000 00001 11000 00000 01110 00100 0?0?0 00000

??010 01001 01011 00000 0000? ??000 10001 00000 00010 00010

00000 00100 0000

*Tanycolagreus topwilsoni*

??10? ????? ????? ????? ?0?0? ????? 0???? 00012 ????? ?????

0?00? ????? ????? ????? ????? ??000 00??? ??1?? 0???? ?????

??010 000?? ????? ?0??0 ????? ????? ??000 00000 0100? 11000

010?? ????? ????? ???1? ????? 10000 00001 00000 00010 00000

0?000 00??0 ????0 ??000 ????0 0???? ????0 ?0??? 00??1 ?0???

????? ?1??? ???11 0??00 10011 11000 00110 00100 01000 10000

01??? ??010 01011 00000 00000 011?? 1?0?1 ??000 00?00 ??0?0

00??? ??0?? 0?00

*Coelurus fragilis*

????? ????? ????? ????? ????? ????? ????? ????? ????? ?????

????? ????? ????? ?0??0 ?1??? ????? ????0 ????1 ????0 000?0

0?010 000?? ????? ??002 ????? ????? ??0?0 ??0?0 0100? 101?0

????? ????? ????? ???1? ???00 10000 00101 ???00 1??10 00?0?

???0? ?0??0 ????1 ?0??0 ???00 0???? ????? ????? ????? ?????

??00? ???0? ?1?11 1???? ???11 1100? ?1?1? ???00 01000 ?????

????? ??010 ?111? 0???? ????? ?11?? 1?0?1 ???00 ??00? ?????

00??? ?000? ????

*Ornitholestes hermanni*

?0110 ???0? 0?00? 1???0 ?010? 01110 0001? 00000 0?00? 10000

0001? 01011 ????? 00000 10000 0?000 000?0 10100 0???0 0011?

01010 0000? ?100? ?0010 ??1?? ????? ????? ????0 ??00? ?????

00010 001?? 010?0 010?0 00101 ?1000 0001? ?0??? ????? 00000

????0 0000? 0010? 00?0? 00?00 01000 01000 11010 ?01?1 1000?

100?0 00?00 00111 0???? ???11 010?0 ????? ???0? ????0 00100

??010 010?0 ?101? 0???? ???0? ???00 00001 00000 ??0?? 1101?

?0000 00000 0?00

*Scipionyx samniticus*

?0110 ????? 0???? ????0 0010? ?0?10 0001? 00?0? 0??00 ??00?

00?0? ????? ????? 00000 102?0 0??0? 000?0 ??10? 0001? 00?3?

?00?? ??0?0 ????? ????? ?0?10 ?0??? ?00?0 000?0 ?00?? 10100

000?0 ??0?0 ????? 0??0? ??000 1?0?? 0000? ????? ????? ?????

????? 000?0 100?? ?0000 ???00 ????0 0??00 1101? ?01?? 10?0?

1?0?0 00?0? 00011 01000 0?010 01000 0?010 101?0 01000 00110

0201? 1?0?0 ????? ????? ????0 000?? ?0?0? 0??00 ??000 110?0

0?0?? 00?00 ?01?

*Juravenator starki*

?0110 ????? 0???? ????0 000?? ?11?0 000?? ?00?0 ?100? 0?0??

?0?0? ????? ????? ?00?0 102?? 0?00? 00010 101?? 00??? ???1?

????0 ?0??? ????1 ????2 000?0 00??? ??0?0 00000 ?00?? ???00

00000 0?0?0 10??? ????? ????? ????? 00010 ??0?? ????0 00???

000?0 10??? ?00?? 00?00 ???00 ??000 ?0000 11010 0010? 100?0

1?0?0 ?0?0? 00??? ?1?00 01010 0?0?0 ??010 ?01?0 01000 00110

000?? ???10 ?1?11 00000 00000 000?? ?0??? 0?000 00000 ??0??

0?0?? ?0000 0010

*Compsognathus longipes*

?0110 ????? ????? ????? 0010? 01110 ?00?0 00000 01?00 0????

????0 0???? ????? 00000 10200 ??000 00010 10101 000?0 00?10

?00?? 0?000 ?0001 ??0?2 00000 00??? ?00?0 00?00 ?10?? ?0?00

000?0 ????? ????0 0100? 100?0 1?0?? 0??1? ??00? 0??10 00000

00000 1100? 00??? ?0?0? ???0? ??000 00000 11010 00100 10000

1?00? ?0?00 00011 01?00 01010 0100? ??010 00??0 ?1??0 ?0?10

??010 01010 ?1011 00000 0000? ?1000 ?0001 0?000 0000? 11100

000?? 00000 0010

*Huaxiagnathus orientalis*

?01?0 ????? ????? ????0 001?? ?1??0 ?0??? ??00? ???0? ?????

????? ????? ????? 00?00 102?? ???0? 00010 1010? 00??0 ???1?

????? ??0?? ????1 ????2 ?0000 00??? ?0000 00000 ?10?? 10100

00000 000?0 ??100 0100? 00000 1?0?? 0???? ??0?? 0?010 00000

000?0 11??0 00??? ?00?0 ???0? ??000 00000 11010 ?0100 10000

1?0?? ?1?0? 00011 ?0?00 00010 01000 0?100 00100 010?0 00000

0?010 0101? ?1011 00000 00?00 01000 ?0001 00000 00000 ?1000

0?0?? ??000 0000

*Sinosauropteryx prima*

?01?0 ????? ????? ????0 001?? ?1110 ?0??? ??000 0??0? ??00?

?010? ????? ????? 000?0 1?2?? ???0? 00010 1010? 00??0 1??10

0???? 0?0?0 ????1 ??112 00000 000?? ??000 00?00 ?10?? 10100

01000 020?? ????0 0100? 10000 1?0?? 00?1? ?000? 0?010 00000

000?0 11??0 000?0 00000 ???0? ??000 00000 11010 0010? 10000

1?0?? ?0?0? 00011 01?00 01000 00000 0?000 00100 01000 00110

00010 01010 ?1?11 00000 00000 ?1000 0??01 00000 00001 11000

0?0?? ?0000 0000

*Deinocheirus mirificus*

????? ????? ????? ????? ????? ????? ????? ????? ????? ?????

????? ????? ????? ????? ????? ????? ????? ????? ????? ?????

????? ????? ????? ????? ????? ????? ????1 ?0000 0100? ?0?10

000?? ????? ????? ????? ????? ????? ????? ????? ????? ?????

????? ????0 ????? ??0?0 ????0 ????? ????? ????? ????? ?????

????? ????? ????? ????0 ?0??1 0?0?? 11000 00100 00000 00000

01??? ????? ????? ????? ????1 100?? ????? ???00 ???11 ??1?0

????? ????? ??0?

*Harpymimus okladnikovi*

?011? ????? ????? ????2 1000? ???00 0???? ??000 0??00 ?????

?0??? ????? ????? 02000 10000 0???? 1?122 ????0 ?0??? 00?11

0001? 00001 ?1000 0?000 00?1? ????? ??0?0 ?0?00 01001 00100

20010 ??000 11??? 0???? ????? ??00? ????? ????? ???00 00001

0??00 00?10 110?? ?1?00 ???00 0?1?2 01100 1101? 00100 ?0010

1?00? 0??00 00011 0??00 ?0??1 0?000 01000 00100 00000 00010

020?? ??0?? ??01? 0???0 00000 10000 ?00?1 ?0000 ?0000 ??110

?00?0 ?0000 ??00

*Pelecanimimus polyodon*

?01?? ?1??? 1???? 1?2?2 1000? ?1001 0?0?? ?0200 0?00? ?????

?1??? ?000? ????? 000?0 100?0 0??0? 00021 ??000 1???1 0011?

00??? ?0??? ????? ????? ????? ??010 0???0 2???2 ?10?? 00110

200?? ????? ????? ????? ????? ????? ????? ????? ????? ?????

????? ?0010 10??? ?1?1? ???0? ??1?1 0?100 11002 0010? 10010

1?0?? 00??1 0001? ????? ??0?1 0?00? 11000 11100 00100 00120

02??? ????? ????? ????? ????1 100?? ?0?0? ????? ??011 111?0

??0?0 00??0 ?10?

*Shenzhousaurus orientalis*

????? ????? ????? ????2 10001 ?10?? 0??1? ??000 ??00? 000?0

0???? ????? ???0? 00000 100?? 0??0? 1?122 ??1?? ????? ?????

0???? 0???0 ???00 000?? ?0??? ????? ????? ????? ????? ????0

20010 000?? 110?0 0100? 00000 0100? 00001 ?0??? ????? ?????

????? 0??1? ?1?00 11??? ???0? ?0102 0?100 ?10?1 00?0? ?00??

??0?? ???0? 0??11 00??? ????? ????0 ??0?? 0?10? 0010? 0010?

0200? 0?0?0 ????? ????? ????1 00?0? ?0?0? 00??? ??0?? ?11?0

??0?0 ???00 ??0?

*Archaeornithomimus asiaticus*

????? ????? ????? ????? ????? ????? ????? ????? ????? ?????

????? ????? ????? ????? ????? ????? ????? ????? ????? 00011

00010 00001 ?1000 00000 ?0??? ????? ??010 201?2 0100? ?0?10

100?? ?000? 11000 11001 ?0020 01000 00001 ?0001 00010 00001

?3?00 00??0 1??10 ??1?1 ????0 00??? ????? ????? ????0 ?????

????? ???0? ??111 0??00 ??0?1 0?0?? 110?0 00100 0?1?? 00120

0??00 01010 0?11? 0???? ????? ?0000 ?0001 00?00 ???11 ??1?0

01??? ?010? ??0?

*Garudimimus brevipes*

?0110 ????0 1101? ???02 10011 01001 000?? 00200 00000 000?0

?0??0 ?0001 ??1?? 00000 10000 02000 1?1?3 ????? ??011 00???

000?? 0?001 ??0?? ????? ????? ????? ????? ????? ????? ?????

???10 00000 1100? ????? ???00 0?000 00001 0000? 00010 00001

??000 0?01? ?1110 ??11? 0010? ???0? ?1100 11012 00100 10010

10000 0???? ?1111 0???? ????? ????? ????? ????? ????? ?????

??0?? ??010 11011 0010? ??00? ????? 000?1 000?? 000?? ?1???

?0000 00000 ???0

*Sinornithomimus dongi*

?0111 ????? 1???? ???02 1001? ?100? 0001? 00200 0000? 00000

0000? ?000? ???0? 00000 10000 0?000 1?1?? ????? ?001? 0011?

00??? 0???1 ?10?? ?0?00 ?0010 0???? ??010 20012 ??0?? 00130

10010 0000? 11000 1100? 00000 01000 00001 ?00?? 00010 00002

03?00 ?0011 1111? 1?111 ???00 00?0? ?1100 11011 00100 100?0

1?0?0 0??0? ?111? ???00 10011 01000 1?000 111?0 00100 00110

02000 01010 1111? 0???0 0??01 10000 00002 00000 ?0011 ?1110

010?? 00100 0000

*Anserimimus planinychus*

????? ????? ????? ????? ????? ????? ????? ????? ????? ?????

????? ????? ????? ????? ????? ????? ????? ????? ????? ?????

????0 ????? ????? ????? ??0?? ????? ???1? ??1?2 ????0 00110

100?? ????? ????? ????? ????? ????? ????? ????? ????? 00002

?3?00 ????1 1??10 ??1?1 ????? ????? ????? ????? ????? ?????

????? ????? ????? ????? ?1??? 0??0? 1?000 11100 01000 00220

01??? ????? ???1? 0???? ????? 100?? ????2 ???00 ???11 ??1?0

????? ??1?? ??0?

*Ornithomimus edmontonicus*

?0111 0?110 ?101? 10102 10011 01?01 00011 00200 00000 00000

01000 0000? ????? 00000 10001 0??00 1?1?3 ????? ?001? 00110

00010 00001 01000 00000 ?0010 01??? ??011 20102 01000 00?10

10010 00000 11000 11001 00000 01000 00001 10001 00010 00002

03?00 00111 11110 1?111 00?00 0??0? ?1101 11012 00100 10010

1?0?0 ???0? ?1?11 00?00 11011 010?0 1?000 11100 01100 00220

02000 01010 ?111? 0???1 00001 10000 ?0002 00000 ?0011 ?1110

0??00 00100 0000

*Struthiomimus altus*

?0111 0?110 ??0?? 10102 10011 01?01 00011 00200 00000 00000

01?00 00001 ?01?? 00000 10001 02000 1?1?3 ????? ?001? 00110

00010 00001 01000 00000 00010 01??? ??011 20102 01000 00110

10010 00000 11000 11001 00000 00000 00001 10001 00010 00002

03?00 00111 11110 1?111 00100 0??0? ?1100 11012 00100 10010

100?0 0??0? ?1111 00000 10011 01000 11000 01100 00100 00110

02000 01010 ?1111 0???1 00001 10000 ?0002 00000 ?0011 ?1110

010?0 00100 0000

*Gallimimus bullatus*

?0111 0?110 11010 10102 10011 01?01 00011 00200 00000 00000

01000 00001 ?0101 00000 10001 02000 1?1?3 ????? ?0011 00110

00010 00001 ?1000 00000 00010 0???? ??011 20102 01000 ?0110

10010 00000 11000 11001 00000 01000 00001 10001 00010 00002

03?00 00011 11110 1?111 00100 00?0? ?1100 11012 00100 10010

10000 0??0? ?1111 00?00 ?0011 01000 11000 00100 00100 00110

02000 01010 1111? 0???1 00001 10000 00002 00000 ?0011 ?1110

01000 00100 0?00

*Falcarius utahensis*

?011? 01000 ??01? 0121? ????? 0???1 ????? ????? ??00? ?????

0?1?0 0000? ????? 20??0 ?0??? ????? 0?001 10000 0???1 0?011

01010 00000 ?110? 11102 ?011? ????? ?0?00 ?0000 11011 m0000

00010 02110 01010 22000 ???01 01000 01001 00010 01010 00000

0?000 00??0 ?0?10 ?00?0 ?0111 11111 1???? ????? 00??1 ???0?

?000? ??100 01011 0?00? ????1 1?01? 01010 00100 01000 00000

0000? ?1011 0?00? 0?0?0 0?000 0101? ?0000 00000 000?0 ??010

?00?? ?000? 0?00

*Beipiaosaurus inexpectus*

????? ????? ????? ????? ????? ????? ????? ????? ????? ?????

????? ????? ????? ?2??1 ????? ????? ???01 00001 ????? ???1?

????? ????? ????1 ????? 1???? ????? ?0??? 0???? ????? 00?00

00?11 0???? ????? ????? ????? ????? 01?0? ???10 ??0?0 00???

??0?? ????? ????? ?0??? ???11 1???1 ????0 ????? ????? ?????

??00? ???0? ????? ??0?? ??0?? ??010 0?110 0010? 010?0 ?100?

0?1?? ???11 ?0??? ?0?1? ????0 ?00?? ????? ?1??? 0?0?0 ??0?0

????? ??00? ??0?

*Alxasaurus elesitaiensis*

????? ????? ????? ????? ????? ????? ????? ????? ????? ?????

????? ????? ????? 220?1 ?0??? ????? ???01 00021 ????? ?????

0?01? 00000 ?1?01 1??02 ?0?1? ????? ????? ???00 0?0?1 00000

00011 021?0 0??10 0210? 0?1?2 ????? 0???? ?00?? ????? ?0000

02?00 00??0 ???1? ?00?0 ???11 ??111 1???? ????? ????1 ?????

??00? ??0?0 11?11 ????? ?0?10 0101? 011?? 00?1? ????? 11000

??1?0 01??? 00??? ????0 0000? ?0?1? ?000? 01000 1?0?? ??000

????? ???0? ??0?

*Erliansaurus bellamanus*

????? ????? ????? ????? ????? ????? ????? ????? ????? ?????

????? ????? ????? ????? ????? ????? ????? ????? ????? ?????

?1??? 0???? ????? ??0?? ????? ????? ????? ???00 ?10?? ?0?00

010?? ????? ?1??? ????? ????? ????0 ??0?? 000?0 00??? ?????

????? ????0 ????? ????? ????1 1???? ????? ????? ????? ?????

????? ???0? ????? ????? ?0?10 010?0 01110 00010 10000 11000

01??? ???01 ????? ????? ????0 100?? ????? ?1?00 ???00 ??0?1

????? 0???? ??0?

*Neimongosaurus yangi*

????? ????? ????? ????? ????? ????? ????? ????? ????? ?????

????? ????? ????? 21??? ?0??? ????? ???0? 000?1 ?1??1 0011?

0101? 0?0?1 ??2?1 1?002 10?1? ????? ?0001 00000 0???? ?????

???1? ?2110 0??2? ????? ????? ????0 0?001 00??? ?0??0 00000

?2010 0???? ?0?1? ??0?0 ????1 1?121 ????0 ????? ????? ?????

??0?? ????0 11011 0000? ??010 ?1??? ????? ????? ????? ?????

????? ???01 0000? 00??0 000?? ????? ?0??0 01110 100?? ???0?

01??? ?000? ???0

*Segnosaurus galbiensis*

????? ????? ????? ????? ????? ????? ????? ????? ????? ?????

????? ????? ????? ?2??1 1?000 0?000 ???01 00021 ????? ?????

????? ????? ???0? ????? ????? ????? ??0?1 0?1?0 0???? ????0

???11 02110 01?20 0210? 0?102 0?0?? ????? ?00?? 0?00? ?0000

?2110 ??00? ???1? ?0?0? ???11 10121 11??? ????? ????1 ?????

??00? ????0 1???? ???02 1?0?0 ??0?? ????? ????? ????? ?????

??100 0100? ?0?01 0011? ??01? ????? ?001? ?11?0 1?0?? ?????

01??? ??0?0 ????

*Erlikosaurus andrewsi*

?0110 ??02? 1?1?0 ???01 00111 00??1 0001? 10000 01000 11000

?0??0 0100? ?1111 22001 00000 02000 1?001 00021 ????? ?????

????? ????? ????? ????? ????? ????? ????? ????0 ????? ?????

????? ????? ????? ????? ????? ????? ????? ????? ????? 0?00?

021?? ??00? ?01?? 00?0? 00011 1?1?1 1?000 1001? 00101 10000

10000 000?0 1???? ????? ????0 ????? ????? ????? ????? ?????

????? ????? ?0?0? 00110 0001? ????? ????0 ??111 100?? ?1???

??010 0???0 0??0

*Therizinosaurus cheloniformis*

????? ????? ????? ????? ????? ????? ????? ????? ????? ?????

????? ????? ????? ????? ????? ????? ????? ????? ????? ?????

????? ????? ????? ????? ????? ????? ??001 000?0 ??0?? 00100

0?0?? ????? ????? ????? ????? ????? ????? ????? ????? ?????

????? ????0 ????? ??0?0 ????1 1???? ????? ????? ????? ?????

????? ????? ????? ???02 ??0?0 0??0? 011?? 00010 1?0?? ?1?10

????? ????? ????? ????? ????? ????? ????? ???11 ???0? ??0?1

0???? ????? ??0?

*Alvarezsaurus calvoi*

????? ????? ????? ????? ????? ????? ????? ????? ????? ?????

????? ????? ????? ????? ????? ????? ????? ????? ????1 ???10

0???? ????? ?2?1? ?2?12 ????? ????? ??0?0 ?0??? ????? ?????

1???0 0111? 0??2? ????? ????? ????? 1?00? ??0?0 1??10 00000

0???0 ????? ???1? ??0?? ????? ???0? ????? ????? ????0 ?????

????? ????? ?1111 10??0 ????? ????? ????? ????? ????? 0?1??

??0?? ????? ???1? ????0 00?0? ????? ?0??1 000?? ?0??? ??11?

?1??? ?000? ???0

*Patagonykus puertai*

????? ????? ????? ????? ????? ????? ????? ????? ????? ?????

????? ????? ????? ????? ????? ????? ????? ????? ????? ?????

??111 ????? ???1? ?2??? ????? ????? ???00 20??3 0210? 110??

110?? ????? ?1??? ???0? ????1 0?00? 11?1? 00??? 10011 ?0??0

????? 01??0 ???1? ??0?? ????0 10??? ????? ????? ????0 ?????

????? ????? ???1? 1???? ????? ??00? 0???? ???0? ????0 0?1??

????? ????1 1??1? ???0? ????? ????? ????? ????1 ????? ??1??

????? ??00? ??0?

*Parvicursor remotus*

????? ????? ????? ????? ????? ????? ????? ????? ????? ?????

????? ????? ????? ????? ????? ????? ????? ????? ????? ?????

???11 0???? ???1? ?2??? ????? ????? ????? ????? ????? ?????

????? ?1?1? ????? 0???? 0??03 ??0?? 11010 0011? 10211 00002

0??00 0???? ???1? ????? ????? ????? ????? ????? ????? ?????

????? ????? ???11 1???? ????? ????? ????? ????? ????? ?????

???0? ??011 1111? 0???0 0000? ???00 ??002 ????? ?0??? ?????

?1??? ??10? 0??0

*Mononykus olecranus*

????? ?00?? ????? 112?? ????? ????? ????? ????? ????? ?????

????? ?100? ????? ????? ????? ????? ???2? ??0?? ????? 01?1?

11101 0011? ???1? ?2??? ???1? ??100 0?000 20003 0210? 1122?

1100? ?1??? ?1??0 00??? ????3 ??0?0 11010 01110 11211 00002

0?000 01??0 0???1 ??0?0 ????0 1??00 0???? ??0?? ????0 ?????

????? ???0? ?0?11 1??00 ?0000 0000? 01000 1000? ????0 0?1??

????? ???11 1??1? ?0000 0000? ???0? ?00?? ?0011 00?1? ??1?0

0???? ?010? 0100

*Shuvuuia deserti*

?0110 10000 0000? 11201 0000? 0???? 0?1?? ??000 0100? 010?0

00010 010?? ?1?11 00010 10000 02100 ??021 ??100 ??011 01110

11101 ?0??1 ?2011 ?2012 00010 0??0? 0?000 20003 021?? 11220

11000 ?1110 11?20 00?00 02103 2?0?? 11010 01110 11211 00002

01000 ?1000 00011 00000 01000 101?2 00100 110?? 00100 10000

11010 0??01 001?? ???00 10000 00000 ???00 1?000 ??0?0 00110

???0? ??011 ?1111 00000 00001 10000 00002 00011 00012 ?1110

0?0?0 ?0100 0100

*Incisivosaurus gauthieri*

?0110 ???1? ?01?? ???10 00101 01000 1001? 0?010 ??000 11000

?0101 01101 01111 20010 10010 01?1? 02022 ??000 2???? ?????

????? ????? ????? ????? ????? ????? ????? ????? ????? ?????

????? ????? ????? ????? ????? ????? ????? ????? ????? ?????

????? ??00? ?00?? 00?0? ?000? ??001 0?000 11?10 ?010? 10010

11001 0???0 1???? ????? ????? ????? ????? ????? ????? ?????

????? ????? ????? ????? ????? ????? ????? ????? ??0?? 11???

??011 0???0 ????

*Protarchaeopteryx robusta*

????? ????? ????? ????? ?0??? ????? ????? ????? ????? ?????

????? ????? ????? ?0??0 10??? ????? 02022 ??000 2???? 00?1?

????? ????? ????0 1???2 10??? ??0?? ?0??? 1???? ?00?? 10100

00000 2?1?? ????? 2200? 1?10? ??00? 1???0 ????? ????? ?0???

000?0 00??? 10??? ???0? ???00 ??1?1 01??0 ????? ??1?? ?0???

????? ???00 10?11 ?0000 ?0011 11000 ??010 ??10? 01000 00000

00000 01011 ?1?1? 00000 0??00 000?? 00001 00?00 00000 11010

?1??? ????0 0100

*Avimimus portentosus*

?0110 ???10 001?0 0??0? ?1?1? ????? 1?1?? ?1??? ??00? 11??0

?0010 0110? ????? 2?1?? 100?0 ??011 0???3 ????? ??011 01111

01011 0?00? ?100? ????? ????? ????? ???11 ?0000 00??? 110??

???00 ?211? 01??0 ?2000 ???01 01000 01000 00??1 10011 11002

0???0 0??0? ??011 0?000 000?0 0??0? ????0 ????? 10??0 ???1?

01??? 0??0? ?001? 1???? ???11 1??1? ????? 0???? ????? ?????

???0? ??011 ?101? 0???0 1000? ???10 00002 ??000 ?0??? ?????

?10?? 001?0 0??0

*Caudipteryx* spp.

00110 ????? ????? ????0 ?110? 01000 0011? 00013 21000 1?00?

00??? ????? ????? 21010 100?? ???10 031?3 ??0?? 00??? ?0?10

0???? 0???0 ???01 ????2 10?11 1000? ?0010 10000 000?? 10000

00010 020?? ?11?0 2200? 11101 0110? 11?00 ??0?? ??010 00002

00000 00000 10?11 0?000 ???0? 00?0? ?1000 11010 00110 10011

1?001 0??1? ?0011 10?10 ?0001 11010 0?010 00000 010?0 00110

1?000 01011 ?1011 00000 0000? ??010 00001 00000 00000 11020

1?0?? 00001 0100

*Microvenator celer*

????? ????? ????? ????? ????? ????? ????? ????? ????? ?????

????? ????? ????? 21?10 ?0??? ????? ????3 ????? ??011 ???1?

???11 110?? ?1??? ?1?02 ????? ????? ???10 1???0 0001? ?0??0

0??10 02?1? 0111? ???0? ???01 01100 01010 00?00 10010 ?????

??0?? 0???0 ?0?11 ??0?? ????0 0??0? ????? ????? ????? ?????

??00? ???1? ?0011 1???? ??011 110?? 0???? ???0? ????? ?0?01

??0?? ??011 ????? ????? ????? ????? 00??? ?0?00 ??0?? ??0??

????? ?0??1 ??0?

*Hagryphus giganteus*

????? ????? ????? ????? ????? ????? ????? ????? ????? ?????

????? ????? ????? ????? ????? ????? ????? ????? ????? ?????

????? ????? ????? ????? ????? ????? ????? ????? ????? 10000

001?? ????? ????? ????? ????? ????? ????? ????? ????? ?????

????? ????0 ????? ????? ????? ????? ????? ????? ????? ?????

????? ????? ????? ????? ????? ???0? 01110 00000 01000 0?001

00??? ????? ????? ????? ????0 ?11?? ????? ????? ????0 ??0?0

????? ????? ??0?

*Elmisaurus rarus*

????? ????? ????? ????? ????? ????? ????? ????? ????? ?????

????? ????? ????? ????? ????? ????? ????? ????? ????? ?????

????? ????? ????? ????? ????? ????? ????? ????? ????? ?0??0

1?1?? ????? ????? ????? ????? ????? ????? ????? ????? 10001

0??00 ????0 ????? ????? ????? ????? ????? ????? ????1 ?????

????? ????? ????? ????? ????? ????? 0???? ??000 01000 0?0??

00??? ????? ??01? 0?000 0010? ?11?? ????1 ??0?? 00??0 ??10?

?1??? ??1?? 0?00

*Chirostenotes pergracilis*

????? ???01 ?0110 1??0? ??1?1 1??0? ????? ????? ????? ?????

????0 1110? ???0? ????? ????? ????? ??1?? ????? ????? ???11

01?11 ????1 ?12?? ????? ????? ????? ???10 1???? ????? ????0

00110 021?0 001?0 22000 1110? 00?0? ????0 ????? 10?10 0?001

00000 ????0 ???1? ??0?0 ?00?? ????? ????? ????? ????1 ?????

?1??? ???0? ?1?1? ????? ??0?? ????0 0???? ??000 010?0 ?1001

000?0 01?1? ?101? 00000 00100 01110 000?1 ?0000 00??0 ??0??

????? ?001? 0?00

*Oviraptor philoceratops*

?0110 ????? ????? 1??00 ????? 111?0 ?011? ??013 2??0? 1?0??

?0?10 0???? ?1??? ?1120 ?0110 0?01? 1?1?3 ????? ?0??1 ?????

????? 1?0?? ????? ????? ????? 1???? ?11?1 ?0?00 0???? 110?0

00??? ?21?0 0???? ????? ????? ????? ????? ????? ????? ?????

????? 00100 100?? ???00 ?000? ????? ??01? 1?010 ??1?? 1?00?

010?1 0???? ?0011 1?010 ?00?1 1?01? ?1010 00100 00000 ?0001

00??? ????? ????? ????? ????1 000?? ????? ????? ??100 ?10?0

1?0?1 0???1 ?10?

*Rinchenia mongoliensis*

?01?0 ????0 ????? ????? 0111? 1???? 1011? 10013 ??00? ??000

?0??? ???00 ??1?2 21120 ?0110 0??11 1?1?3 ????? ?1??? ?????

?1??? 1???1 ???0? ????? 1???? ????? 111?1 ??000 00??? 1?0??

00?10 ??1?? 0???? ????? ????? ????? ????? ?0??? ?0??? ?0???

????0 ??10? ?001? ???00 ???0? ????? ???10 1?1?? ?01?? 1110?

010?1 0???? ????? ????? ???1? ????? ????? ????? ????? ?????

??0?? ????1 ????? ????? ????? ????? ????? ?0??? ??1?? ?1???

??111 00?11 ????

*Citipati osmolskae*

?0110 01001 001?? 22100 01111 11000 1011? 10013 21000 11000

00110 01100 01102 21120 ?0110 01011 1?1?3 ????? ?1011 00110

01011 11001 ??20? ??002 10011 1?011 111?0 10000 000?1 10000

01010 02?10 0?110 220?0 ??101 0010? 11010 00000 100?0 00001

00000 00100 10011 0?000 00000 0??0? ?1010 11110 10101 11101

01001 ???0? ?10?? ??010 ?0010 11010 01010 00000 00000 00001

00??? ???1? ?101? 00000 00101 10010 00001 ??000 00100 ?10?0

1?111 0?0?1 0100

IGM 100/42: "Zamyn Khondt oviraptorine" of Osmólska *et al*. (2004)

?0110 ????? ????? ????0 01111 110?0 1011? 10013 2100? 11??0

00??? 01100 ?1112 21120 ?0110 0??11 1?1?3 ????? ?10?? 0??1?

?1?1? 1???1 ???01 ??002 10?1? ??01? 11111 10000 ?0??? 10000

00110 02110 ?1110 220?? 1?101 0?1?? 1?010 00??? ????? 00001

00000 00100 ?001? 0??00 ?0?0? 00?0? ?1010 11110 1010? 11101

01??1 0??0? ?0010 10010 ?0010 11?10 0?010 001?0 00000 00001

00000 01011 ?1011 0???0 00001 10010 ?0001 00010 00100 ?1020

11111 01011 0100

*Ingenia yanshini*

?01?0 ????? ????? ????? ?1111 11000 10?1? 1??13 2??0? 1???0

?0??0 011?? ???12 21120 ?0110 0?011 1?1?3 ????? ????? 0??1?

?1??? 1???2 ???01 ????2 100?? ?0011 11111 1?000 000?? 10000

01010 0211? ?11?0 22000 01101 0?110 11010 0000? 10010 00000

00000 ?1100 ?0?11 ??000 ?0?00 00?0? ?1010 11110 ?01?1 1110?

010?1 0??0? ????? ?0010 ?0010 11010 01010 00000 10000 00011

00000 01011 ?101? 00??? ??001 00010 00000 00000 00101 ?10?0

11011 00011 0100

*Conchoraptor gracilis*

?0110 ????? ????? 1???0 0111? 1100? 1011? 10013 21000 110?0

00??? ??1?0 ????2 21120 ?010? 0?011 1?1?3 ????? ????? ?????

010?? 1?012 ?110? ?1??? 0?011 1???1 ?11?1 10000 10??? ?????

011?0 02100 01?10 2???0 ???01 001?? 11010 ?0??? ?0??0 00000

??0?0 ???00 ??011 0??0? 00000 0??0? ??010 1111? 1010? 11101

01??? ????? ????? ??0?? ???1? 1???? 010?0 ???0? 00?00 00111

000?? ????1 ???11 0???? ???0? 00010 00000 ????? 0???0 ?10?0

??011 0101? ??0?

*Khaan mckennai*

?0110 ????? ????? ????0 0111? 110?0 1011? 10013 21000 110?0

00??0 0110? ????? 21120 ?01?0 0?011 1???? ????? ?1011 0??1?

?1011 110?? ????1 ??002 10?11 1001? ?1111 10000 ?00?1 10000

01110 02110 ?1110 22000 1?101 ??1?? 11010 0?0?? 10010 00000

00000 00100 10011 0?000 ???00 0??0? ?1010 11110 10101 11101

010?1 0??0? ?0011 10010 ?0010 11010 01010 00000 00000 00011

00000 01011 ?1011 00000 00001 00010 00000 00000 00100 ?10?0

1?0?1 01011 0100

*Heyuannia huangi*

??11? ????? ????? ????? ????? ????? ????? ?0??? ????? ?????

0???? ????? ????? ?1120 ?011? 0??1? ????3 ????? ?1??? ?????

????? ??0?2 ????? ????? ?0?11 1???? ?11?? 10?00 ?10?? 1110?

01010 021?? ?1??0 220?? 01101 ??1?? 1?010 000?0 ?0??? ?0000

000?0 01??0 1??1? ???00 ???0? ???0? ?1??? ????? ????? ?????

??0?1 ???0? ?00?1 10010 ?00?0 1?010 0?010 00000 1?0?0 0?01?

??000 01011 ?1?1? 0?000 ?000? ??010 ?0?00 ?0000 001?1 ??0?0

1?0?? ??01? 0?00

*Anchiornis huxleyi*

00110 0???? ????? ????1 1010? ?111? 000?? ??002 2?00? 1????

01?1? ????? ????? 000?0 11??1 ???0? 00020 ???1? ?0?10 1???0

0?0?? 010?0 1???0 11012 10??0 11??? ?0010 11110 000?? 10000

00000 ?21?1 00110 0200? 1?101 1100? ?1?1? 000?? 1?01? 00012

11000 10??0 10?1? ?00?0 ???0? ???03 00100 11010 00101 100?0

1?0?? ?0??? 01111 11000 10011 12011 01010 0010? 01001 11001

01020 01011 ?1011 00?12 0?110 00010 00?12 1000? 00000 11010

1?0?? 00000 0?01

*Sinovenator changii*

?0??? 1002? 00001 11200 00101 ?1110 0???1 ????? ?0000 11???

011?1 0100? ????? 000?0 ?1??? ???00 00021 ??01? ????0 1?111

00011 01000 01000 110?? ??1?? ????? ??010 111?0 ????? ?????

000?? ?21?? 01110 02000 11101 1100? 11110 000?? ?0010 00102

1??00 0???? ?0?1? ?0000 11?0? ?0??0 01100 ?10?0 00??? 10?1?

1100? ?0?00 00111 1??00 100?? ????? ????? ???0? ????0 00000

???20 11011 ?101? 1???0 011?? 0??10 00102 ?00?? 000?? 1?0??

110?? ?00?? 0???

*Mei long*

?0??? ????? ????? ????1 1010? ????0 0?01? 00002 21000 01?10

?01?? ?100? ????? 000?0 110?? 1?000 0?021 ??01? 00110 11110

011?1 010?0 1???0 11012 ?1110 11??? ?001? 11?10 ?0?1? 10000

000?? ?2111 ?1??? 020?? 11101 110?0 11110 0?010 1101? 00012

11011 ?0?00 00??? 00000 ????0 ????0 ?1100 11??? 001?1 10010

1???? ???0? ?1111 ??000 10011 110?0 ??0?? ???0? ????? 00000

????? ???1? ?1?11 100?1 ?1??0 00?11 ??1?2 1?000 000?0 1?010

110?? ?0?00 0001

*Byronosaurus jaffei*

????? 101?? ?101? 11001 00101 011?0 0???? ??202 20??? ?????

????1 ?100? ????? 00000 11??1 1???? 00021 ??01? 0?0?? ?????

01011 0???? ????0 ??02? ????? ????? ????? ????? ????? ?????

????? ????? ????? ????? ????? ????? ?1??? ?0?0? ?0??? ?????

1???? ????? ?0??? ?0??? ?100? ??0?0 0?100 ?1011 00??? 100??

?10?0 011?? ????? ????? ????? ????? ????? ????? ????? ?????

????? ????? ????? ????? 11??? ????? ????? ????? ??0?? 11???

??0?? ????? ????

*Sinornithoides youngi*

?0??0 ????? ????? ????1 ?000? ?1??? 0???? 00202 2???? ???1?

0???? ????? ????? 00??0 ?10?? ????? 00011 0101? ????? 11?10

01??? ????? ???00 11?12 10210 1???? ??01? 11??0 ?00?? 10000

000?? ????? ????0 ?200? ????1 ?1??? 11110 ????? 1???? 00012

11001 ?0??0 10??? ?0000 ???00 ????3 01100 11011 ??1?1 100??

1?000 ?1?0? 011?? ?0?00 ??011 11010 01010 00100 00000 00000

0???0 ???11 ?1111 10001 11110 00010 ????2 10000 00000 1?010

110?? ?0?0? 0001

*Sinusonasus magnodens*

?0??? ????? ????? ????0 101?? ?111? 0???? ??20? ????? ?????

????? ????? ????? ?00?0 11??? ????? 0?011 10?1? ????? ?????

????? ????? ????0 1??22 00??? ????? ????? ????? ????? ?????

????? ?2??? ????0 02??? ??10? ????? ????? ??0?? 10?1? 00??2

110?1 ????? ?0??? ?0??? ???0? ????? 0?100 11011 001?1 100??

??1?0 ????? 0???? ?0??? ????? ????? ????? ????? ????? ?????

????0 11?11 ?1?11 ?0001 1110? ???1? ??102 1?0?? 000?? ?1?1?

??0?? ??100 0??1

*Troodon formosus*

???1? 1112? 11010 00001 ???0? 01010 0???? ??202 20000 210?0

0?1?0 1100? ???0? 10??0 ?1??? ????? ??011 01010 0???1 11110

01011 01111 ?1000 ?1022 ??11? ????? ????? ????0 10??? ?0???

????? ??1?? ????? ????? ????1 ??0?0 11?10 0???? ??010 00012

10?01 ????0 ?0??? ?0??0 1000? ??0?3 0???? ??0?1 ????1 ???10

?000? ??1?1 0??11 1???? ????? 1?01? 0???? ????? ????? ?????

????? ????? ??11? 10101 1110? ????? 0?101 ??0?? 000?? 0????

?10?? ?01?? 0??1

*Saurornithoides* *mongoliensis*

?01?? 1?1?? 1101? ??0?1 00001 ?1000 0???? ????2 ????? ?????

????? ????1 ?010? 100?0 110?? 1???? 00011 01010 ????? ???1?

??0?? 0???? ?100? ?1??? ?0??? ????? ????? ????? ????? ?????

????? ????? ????0 02000 ?0101 ?10?? 11110 ????? ????? 00??2

11?01 ????? ?0??1 ?0??? ??00? ?00?3 0?100 11011 00101 000??

1?0?? ?1??1 0??1? 10??? ????? ????? ????? ????? ????? ?????

???00 110?1 ???1? ?0101 1110? ???10 0??0? ??0?? ??0?? 01???

??000 ??10? 0??1

*Zanabazar* *junior*

?0110 1?12? 11010 0?001 1000? ?100? 000?? ??202 2000? 21??0

????1 1100? ????? 100?0 11??? 1???? 00011 01010 0???? ?????

????? ????1 ?1000 ?1022 ????? ????? ????? ????? ????? ?????

????? ????? ????? ????? ????? ????? ????? ????? ??01? ?????

????? ????? ?00?? 00??? 1100? ??0?3 0?100 11011 0010? 00010

10000 ?11?? 0???? ?0??? ????? ????? ????? ????? ????? ?????

????? ????? ???1? ????? ????? ???1? ????? ????? ??0?? 01?1?

??0?0 ??1?? ????

*Unenlagia* spp.

????? ????? ????? ????? ????? ????? ????? ????? ????? ?????

????? ????? ????? ????? ????? ????? ????? ????? ????? ?????

??111 1000? ????? ????? ?0??? ????? ??0?0 ??110 1???? ?????

???01 22111 01111 20010 ???01 11001 1?110 000?? 10?10 ?0112

1?000 0???? ???11 ????0 ????0 00?0? ????? ????? ????1 ?????

????? ???0? ???10 1???0 ?0?11 ????? ????? ????? ????? 1?0??

??02? ??011 ?101? 0?0?0 111?? ???12 211?? ?0000 0???? ?????

1???? ????? 0?01

*Buitreraptor gonzalezorum*

?0110 ????? ????? ????? 1???? ?11?? 000?? ?0??? ??000 00??1

0011? 0???? ????? 00??0 11??? 0???? ??021 ??100 ?0010 01110

01111 0000? ?1000 11012 ?021? ????? ?0010 11110 00?1? 100?0

????? ?1110 01??1 22??0 1110? 110?? ?1110 000?? 10010 ?0112

1?000 0???? ???1? ?00?0 ???00 00?00 00??? ???10 0010? 1?010

1?00? ???00 ?1111 0?000 ?0011 120?? ????? ????? ????? ?????

??021 0101? 0101? 0?010 01??? ???12 01102 1??00 0?0?? 1??1?

0?0?? ?0000 ????

*Rahonavis ostromi*

????? ????? ????? ????? ????? ????? ????? ????? ????? ?????

????? ????? ????? ????? ????? ????? ????? ????? ????? ?????

01111 1?0?1 ?11?0 11012 ?02?? ????? ??0?? ??11? ?001? ?????

???01 12110 01111 22?00 1?101 ??001 11?10 000?? 1??10 00110

11000 ????? ???1? ????0 ????? ?0?0? ????? ????? ????? ?????

????? ???0? ???11 11??3 ?0??? 130?? ????? ????? ????? ?????

??021 ??011 ?101? 00?10 021?? ???11 ?11?1 100?? 01??? ???1?

11??? ??00? 0??1

*Bambiraptor feinbergorum*

?0110 ????? 0?0?1 ??1?? 00?0? 01110 000?? 10201 ??1?? 1????

0001? 0???? ????? 000?0 10??1 ??000 0?010 10100 ??110 0?120

01111 100?0 ??100 11??1 10??? ??0?1 10010 11110 1001? 10100

0001? ?2110 01110 02000 11102 11000 1?110 00000 10010 00110

1?000 00?00 ?0?1? ?0000 ????0 00?00 00000 ?1013 001?1 10010

1?10? 11100 00110 11000 ?0011 12010 11010 00100 00010 11001

01020 11011 01010 00000 01100 ?0012 00101 10000 01000 01011

110?? ?0000 0101

*Sinornithosaurus millenii*

?011? ????? 0???? ????? ?00?? ?1110 0???? 10201 ?1100 11???

0?01? ????? ????? 00000 100?? 1???? 01010 11100 3???? ???1?

?1??? ????0 ???00 ?1??1 ?0??? ??011 1?010 31110 ?00?? ?0?00

00010 ?211? 0??10 02?0? 11102 2?0?? ??1?? ????? 1?010 00112

110?0 ?000? 1001? ?0000 ???0? ?00?0 00100 11013 00?01 10010

1?100 0110? 0???? ??000 10011 121?0 01011 00001 00011 11000

01?21 1111? ?1?10 00012 01?00 00012 ??112 ?0000 01000 01010

1?0?? 0?0?0 0101

*Microraptor* spp.

10110 ????? ????? ????? 00??? ????? ????? ????? ????? ?????

?0??? ????? ????? ?0??0 10?0? 1???? 0?010 0000? 3???0 01?1?

?01?1 000?0 1???0 110?1 1?211 1?01? 100?1 31?10 100?1 10100

00110 12110 ?0110 0200? 11102 2100? 11111 ??00? 1001? 10112

11000 00??0 10?11 ?000? ???00 ?00?0 0?100 ?1??? ????1 ?00??

1?1?0 ???0? ?0?11 11000 10011 12110 ??011 00001 00011 11001

01021 11110 ?1110 00012 01110 00002 0?112 10000 01000 1?010

1?0?? ?000? 0101

NGMC91: unnamed dromaeosaurid described in Ji *et al*. (2001)

?011? ????? ????? ????2 00??? ?11?? 00??? ???01 ???0? ?????

????? ????? ????? ?0000 100?? 1??0? 0?010 ??10? ?0??? ?????

????? ????? ????0 1???1 ????1 ????? 10??0 31?10 ?00?? 1?100

001?? ????? ????? ????? ????? ????? ???1? ??0?? ????? ?????

11??? ????? 10??? ?000? ???00 ????0 00100 11013 ?01?1 100??

1?1?0 ?1?0? 00??? ??0?0 ?0011 12110 ??011 000?1 00011 01001

01??? ???1? ?1?1? ?0012 01110 000?? ????2 1?000 01000 11010

??0?? ????0 ?101

*Tsaagan mangas*

?0110 01001 00001 20112 00001 01010 0001? 10201 21111 10001

00011 00001 ?0??? 00000 100?1 11100 01010 10100 ??1?0 0?12?

????? ????? ????? ????? ????? ????? ??010 ????? ????? ?????

????? ????? ????? ????? ????? ????? ????? ????? ????? ?????

????? ???0? ?00?? ?0000 0000? ??0?0 0?000 11013 0000? 00000

101?? 10??? 0?1?? ???00 ??0?? ????? ????? ????? ????? ?????

????? ????? ????? ????? ????? ????? ????? ????? ??0?? 01???

??0?0 00??0 ????

*Adasaurus mongoliensis*

??110 0???? ????? ????? ????? ????? ?0??? ??201 ????? 1????

?0??? ?0?0? ????? ????? ??0?? ???0? 0?0?? ????? ?01?? ?0?2?

01?1? ????0 11100 ????1 ????? ????? ??0?1 1?1?? ????? ?????

???00 22110 01110 ???0? ???02 1?0?? 1???0 0???? ????? 10110

??001 0???? ?0?1? ?000? ????? ?0?00 0???? ????? ??0?1 ?????

1???? 0???? ?011? 1??00 ?0??? ????? ????? ????? ????? ?????

??02? ??011 ?1011 00010 1110? ???12 ???01 ?00?? 01??? ???1?

??0?? ??0?0 ???1

*Velociraptor mongoliensis*

?0110 01001 00001 20112 00001 01110 00011 10201 2111? 10001

00010 00011 10101 00000 10011 11100 01010 10100 30110 00120

01111 10110 11000 11011 10211 11010 10011 11110 10011 10000

00000 22110 01110 02000 11102 11001 11100 00000 10010 00110

10010 00000 ?0011 00000 00000 00000 00000 11013 00001 00000

10100 10100 00110 10000 10011 11010 01010 00100 00000 11000

02020 ??011 0001? 00000 11100 00012 00001 10000 01000 01010

11000 0?000 0101

*Deinonychus antirrhopus*

?0110 ????1 ????? ??11? 0000? 01110 00011 10201 2???? ??0?1

????0 ?00?1 1010? 000?0 ?0011 1?100 0?010 10100 3?110 00120

01111 1011? ???00 11011 0?21? 10??? 1?010 11110 10011 10000

00010 22110 01110 02000 1110? 01001 11110 00000 10010 00110

10000 00?00 ?0011 00000 ?0?00 0?000 0??00 ??013 000?1 00???

1000? 1?100 00100 1???? ??011 11010 01010 00100 00000 11001

02000 01011 00011 00010 11100 00012 ?0000 10000 01000 01010

11000 00000 1?01

*Achillobator giganteus*

????? ????? ????? ????? ????? 01110 ????? ????? ????? ?????

????? ????? ????? ????? ????? ????? ??000 101?? ????0 ?0120

0?111 101?? ????? ??011 ??1?? ????? ??010 1???? ????? ????0

0?010 220?0 11110 0100? 01101 0?00? 11110 ?0??? ?00?0 00?10

1???? 0???? ???1? ??0?? ????? ?0?00 0???? ??013 ????? ?????

????? ??10? ?0?00 ???00 1?0?? ?1??? ????? ???0? ????0 1?0??

??000 01001 00?1? ????? 11??? ???10 00000 ?0??? ????? ??0??

1???? ?0??? ??0?

*Dromaeosaurus albertensis*

?0??0 01000 00000 0010? ?0?0? 0???0 ??011 10??? ?1111 ????1

0001? 10011 00??? 00000 10011 11100 0?000 10100 3???? ?????

????? ????? ????? ????? ????? ????? ????? ????? ????? ?????

????? ????? ????? ????? ????? ????? ????? ????? ????? ?????

1???? ??00? ????? 00?0? 0000? ??0?0 0?000 ??0?? ??0?? 00?00

10010 100?0 0???? ????? ????? ????? ????? ????? ????? ?????

????? ????? ????? ????0 111?? ????? ????? ????? ??0?? 01???

??000 ????0 ????

*Utahraptor ostrommaysorum*

????? ????? ????? ????? ?0?0? ????? ????? 10000 ????? ?????

????? ????? ????? ??0?? ??0?1 ????? 01?0? 101?? 3???0 ?????

??111 ???1? ????? ???0? ???1? ????? ????? ????? ????? ????0

????? ????? ????? ????? ????? ????? ????? ????? 10010 ?????

1???? 0??0? ????? 0???? ????? ????0 ????0 ??0?? ????1 ?0???

????? 100?? ???00 1???? ????? ????? ????? ???0? ????? ?????

????? ????? 0???? ????? ???0? ????? ????? ????? ????? 0??10

????? 00??? 1???

*Epidendrosaurus ninchengensis*

?0??? ????? ????? ????? ????? ????? ????? 10000 ?0?00 ?????

????? ????? ????? 20000 10000 0?000 ????? ????? ????? ?????

??00? 010?0 0???0 1?000 11??? ?1??? ???10 11?10 ?00?? 10100

00001 1?1?? ?1??0 20?0? ??111 2?0?? 1??10 0?0?? ?0??0 00001

010?0 00?01 10?1? ?000? ???00 ?0?0? ?0??? ????? ??1?1 ????0

1?0?1 0??0? 00?11 11??? ?0021 02010 ??010 00100 00000 00000

000?? ??011 ?1?1? 01102 00001 000?? ?0001 10000 00010 ?1010

1?0?? 1?000 0?00

*Epidexipteryx hui*

?0??? ????? ????? ????? ????? ????? 00??? 000?? 00?00 11???

?000? ????? ????? ?110? 1?0?? ??000 01020 ??100 00??? ?????

?0??? 0?0?1 ???10 2??12 3??10 0?1?? ??010 1?010 00??? ????0

0??01 1?10? ????0 20?0? ?1110 2?0?? ????? ??0?? ??011 10???

?10?? 00??? 10??1 ?1?0? 1??00 0???1 10??0 11??? ????? ????0

1?1?? ?0?0? 00?11 1??00 ?0021 120?? ???1? 0???? ????0 00000

??00? ??01? ?1011 0???? ????? ???10 ?0001 10?00 0?0?? 1?0??

11??? 100?0 ?00?

*Archaeopteryx* (without data from London specimen)

10110 0?0?? 000?1 1???1 1010? ?1110 0?011 00000 20000 00010

0?00? 01001 11?01 000?0 11200 02000 00020 ??000 0001? 01?10

000?? 0???0 ???00 21012 11200 01??? ?0010 11110 ?00?? 10000

00001 121?0 001?0 0200? 1?101 1100? 11110 00?0? 10010 00001

01000 00000 10?11 ?0000 ???00 00102 00101 11010 00101 10010

1?00? 00?00 01?11 11010 ?0121 13011 0?010 00100 01000 01001

02021 10011 ?1011 01112 00110 00010 00112 10000 00000 11010

1?00? ?000? 0000

*Wellnhoferia grandis*

?0??? ????? ????? ????1 10?0? ????? ????? ????? ????? ?????

????? ????? ????? 00??0 112?? ????? 00020 ??00? 0???? ?????

????? ????0 ????0 2?002 31??0 0???? ??010 11110 ?0??? ?0?00

00001 ??1?? ??1?? 0??0? 1?101 1?01? 1?110 0?0?? ??010 0???1

010?0 00??0 10??? ?00?0 ???00 00?02 00101 ?1??? ????1 ?00??

??0?? ?0?0? 0??11 ?1?10 ?0?21 130?1 0?011 00100 01000 01001

0202? ??011 ?1?11 01112 00??0 0001? ?0?12 10000 00000 1?010

1???? ???0? ?000

*Jeholornis prima*

10??? ????? ????? ????1 ????? 0???? ????? ?0??0 ???0? ?????

????? ????? ????? 120?0 11??0 0200? 1?1?2 ??0?? ?0??? ?????

????? ????1 ????0 2???2 1121? ???1? 1?010 3?110 ?00?? 11000

00001 221?? 0???1 0??0? ???02 1?0?? ???1? 000?? 1?010 11??2

010?0 00?00 10??? ?1000 ???00 ?0102 ?0?0? 1???? 0?101 1???0

1?01? 0??0? 01?11 11?13 10121 13011 ??011 000?1 10011 00000

0002? ??01? ?1?1? 01112 00010 0101? ?0111 10000 00000 ?1010

1?0?? 1??00 0100

*Sapeornis chaoyangensis*

?011? ????? ????? ????1 1011? ????0 0001? 00000 2??00 01???

00??? ????? ????? 000?0 112?0 0?000 00??3 ????? ?0??? ?????

0?00? 010?2 ???0? ????? 21?10 01??? ?1000 11110 ?00?? 11200

00?01 1?1?? 01??1 00?0? 1?102 1101? ???10 000?? ?0??1 11002

0???0 00?00 10?1? ??000 ???10 10?0? ?0?0? 1101? 00101 ?0??0

1?0?? 0??0? ?1?11 1?013 10121 13111 ?1011 100?1 11001 1100?

1?02? ??011 ?1?11 0111? ???1? ?101? 00?01 ?0000 00000 110?0

110?? 1?00? 0100

*Confuciusornis sanctus*

10110 ????? ????? ????1 1000? 0???0 0001? ??0?0 2??0? 01??0

?0??? 01??? ????? 00010 100?1 0?000 1?1?3 ????? ?0??? 1??1?

???01 110?2 ???0? ????? 2??11 1?110 10001 31110 00011 11000

00101 12011 ????1 00?00 12103 21011 1??10 ??11? 11211 11011

010?0 00000 10011 0?000 ???00 1??0? ?0001 ?1??? 00101 000?0

1?0?1 0??0? ?1111 1?010 10121 12111 01010 00001 01010 11001

0002? ??011 ?1?11 01112 00010 1101? ?0111 ?0000 00000 ?10?0

1?0?? 100?0 0100

*Protopteryx fengningensis*

10??? ????? ????? ????1 10??? ????? ????? ??0?? ????? ?????

01??? ????? ????? 000?? 1???? ???0? 0??2? ??0?? 0???? ?????

0???? ?1??2 ????? ????? 2??11 1?11? 11??0 31?10 000?? 11200

00001 ????? ????? ???0? 1?1?? ????? ???1? ??1?? ????? ?1112

010?0 ?0??0 101?? ??00? ????0 ????2 ?01?1 11??? 00?11 ?00?0

1?0?? ????? 01??? ??1?3 ?0121 13111 ??011 000?1 10010 11000

1???? ???11 ?1?1? 01112 00?0? ?10?? ????1 ??000 00000 110?0

1?0?? ??0?? ?100

*Yanornis martini*

?01?? ????? ????? ????1 11?0? ????? 0???? ??0?? ????? ????0

?1?0? ????? ????? 000?0 112?0 0???? 00020 ??000 0???? 11???

????? ??0?2 ????? ????? ???11 1?1?? ?0?00 31?10 ?00?? 1120?

00??? ????? ????? 00??? 12103 2?01? ??110 0?1?? 11??1 12?0?

010?0 00??0 10??? ?00?? ???00 1?1?2 00001 ?0??? 00111 ?00??

1?00? 00?00 01111 ??1?? ?0121 13111 ??011 0?001 1?0?1 11110

1???? ??01? ?1?11 01??2 0000? ??0?? ????1 ??000 00000 110?0

??0?? 10??? 0100
